# Regular antenatal care visits were associated with low risk of low birth weight among newborns in Rwanda: Evidence from the 2014/2015 Rwanda Demographic Health Survey (RDHS) Data

**DOI:** 10.12688/f1000research.51969.1

**Published:** 2021-05-19

**Authors:** Emmanuel Biracyaza, Samuel Habimana, Donat Rusengamihigo, Heather Evans

**Affiliations:** 1 Department of Community Health, University of Rwanda, Kigali, Rwanda; 2 Sociotherapy Program, Prison Fellowship Rwanda (PFR), Kigali, Rwanda; 3 Executive Director, Rwanda Resilience and Grounding Organization (RRGO), Kigali, Rwanda; 4 Department of Clinical Psychology, University of Picardy Jules Verne, Amiens, France; 5 Evans Counseling Services, Coopersburg, USA

**Keywords:** Antenatal care; Birth weight; newborn; Maternal factors; Sustainable Development Goals; Pregnancy

## Abstract

**Background:** Low birth weight (LBW) remains the global unfinished agenda in most countries of the world especially in low- and middle-income countries. LBW subsequently has harmful effects on the lifestyle, psychosocial and physiological development of the child. Although it is known that antenatal care (ANC) visits are important interventions contributing to prediction of newborn birth weight, little has been conducted on effect of ANC visits on birth weight in Rwanda. This study aimed at determining the association between regular ANC visits and risk of LBW among newborns in Rwanda.

**Methods: **A cross-sectional study design was conducted to analyse the effects of ANC on LBW using the 2014/2015 Rwanda Demographic Health Survey. Associations of socio-demographic, socio-economic, and individual factors of the mother with LBW newborns were performed using bivariate and multiple logistic regression analyses.

**Results**: Prevalence s of LBW and macrosomia were 5.8% and 17.6%, respectively. Newborns delivered from mothers attending fewer than four ANC visits were at almost three-times greater risk of having LBW [aOR=2.8; 95%CI (1.5–5.4), p=0.002] compared to those whose mothers attending four or more ANC visits. Residing in a rural area for pregnant women was significantly associated with LBW [aOR=1.1; 95%CI (0.7–1.6), p=0.008]. Maternal characteristics, such as anemia, predicted an increase in LBW [aOR=3.5; 95%CI (1.5–5.4), p<0.001]. Those who received no nutritional counseling [aOR=2.5; 95%CI (2–8.5), p<0.001] and who were not told about maternal complications [aOR=3.3; 95%CI (1.5–6.6), p=0.003] were more prone to deliver newborns with LBW than those who received them. Pregnant women who received iron and folic acid were less likely to have LBW newborns [aOR=0.5; 95%CI (0.3–0.9), p=0.015].

**Conclusion**: ANC visits significantly contributed to reducing the incidence of LBW. This study underscores the need for early, comprehensive, and high-quality ANC services to prevent LBW in Rwanda.

## Introduction

Antenatal care (ANC) globally is an important health strategy that has been considered as an essential intervention to contribute to the health of newborns and their mothers. This health intervention reduces low birth weight (LBW) which is a foremost public health burden that weakens the development of families and nations due to its harmful effects on quality of life. ^1^ LBW is defined when a baby weighs less than 2.5 kilograms at birth. This health burden particularly, in low- and middle-income countries (LMICs) may cause multiple effects, such as birth asphyxia, amniotic fluid aspiration, hypoglycemia and hyponatremia. ^2^^–^^4^ The estimate of 15.5% to 15.9% of LBW worldwide was reported ^5^^,^^6^ and the high prevalence (95.6%) of LBW newborns occurred in LMICs. ^7^^,^^8^ ANC visits were found to play a great role in the health promotion of mothers and children before, during and after delivery. It specifically contributes to the reduction of morbidity and mortality of mothers and children. ^9^^,^^10^ Epidemiological studies have shown that the children weighing less than 2.5 kilograms are nearly 20-times more likely to die than those who weigh 2.5 kilograms and more. ^11^ Other studies have stated that newborns with LBW were 5–10-times more likely to die than those who weighed 2.5 kilograms and more. ^2^^,^^3^ Prior studies have indicated that the newborn may develop macrosomia, defined as birth weight more than 4 kilograms. These newborns are considered to have an excessive birth weight. Its prevalence varies globally between 3 and 15% depending on the region, due to various factors where it remains higher in the developing world. ^12^^,^^13^

Prior studies indicated that maternal and environmental factors are major factors with regard to LBW. ^14^^,^^15^ The number of ANC visits attended by the pregnant women and the amount of recommended packages of the interventions provided during ANC are the main factors contributing to LBW. ^14^^,^^16^ A low socio-economic status and limited access to qualified health services related to pregnancy are risk factors for LBW. ^16^ Multiple studies established that accessibility to poor food consumption behaviors, problems of health status for pregnant women, low caloric intake, hypertension, urinary infections, smoking behaviors, genital infections and psychological distress were found to be risk factors of LBW. ^4^^,^^17^^,^^18^ Earlier studies conveyed that consumption of iron, birth order, and calcium supplementation reduces the risk to develop LBW. ^18^^–^^20^

ANC refers to the routine health management of presumed healthy pregnant women without the symptomatology or screening so as to diagnose and detect the diseases or complicating symptoms. ^16^^,^^21^^,^^22^ The World Health Organization (WHO) recommended pregnant women attend a minimum of four ANC visits for obtaining the basic health education and interventions that promote health of babies. The more they attend ANC services, the more they receive the maximum health package that plays a crucial role in their health and the health of their newborn. ^23^ Prior studies have documented that two-thirds of all pregnant women worldwide attended at least one ANC service during pregnancy. They also documented that ANC is the significant predictor of birth weight where attending the required ANC increases the probability of achieving full life-saving potential and having a newborn with more than 2,500 grams. ^24^^–^^27^ In ANC visits, pregnant women are offered health education, such as adequate nutrients to be consumed during and after delivery, vitamin intake, proper vaccination, changing risk behaviors such as smoking, and place of delivery. ^28^^,^^29^ The package provided to pregnant women consists of many interventions like the identification and management of the obstetric complications including pre-eclampsia, tetanus toxoid immunization, de-worming, iron, folic acid, and interventions related to infectious diseases, such as malaria prevention, during pregnant period and insecticide-treated nets (ITNs). The fundamental rudiments of a focused approach to ANC are surveillance of health issues among pregnant woman and their babies, management of complications occurring in the pregnancy period, such as hypertension, treatment of underlying or concurrent illness, screening for health conditions and diseases, mental health problems and intra-partner violence or domestic violence. ^25^^,^^29^^,^^30^ ANC visits are obviously the opportunities for promoting the use of skilled attendance at birth, injury prevention, adherence support for preventive interventions, healthy lifestyle safety, and healthy behaviors such as breastfeeding, early postnatal, and planning for the optimal pregnancy spacing. ^24^^,^^31^^,^^32^ Pregnant women are provided supplementary nutrients for enhancing the baby’s and mother’s health, screening for genetic and congenital disorders, and offered folic acid supplementation to reduce the risk of neural tube defects. ^1^^,^^8^^,^^28^^,^^33^^,^^34^

Earlier researchers documented that through ANC services, pregnant women and their newborns develop physiological and psychosocial wellbeing. It also reduces risk to have a LBW newborn. ANC visits contributed to development of healthy behaviors and compromise of the emergency preparedness plan to intensify the maternal awareness, improving the newborn needs and self-care. ^29^ Other studies indicated that ANC visits contribute to weight gain and weight regulation of women. They confirmed that ANC services increase the weight of pregnant women and contribute to the growth of the fetal and maternal tissues and fluids. ^21^^,^^35^ This health intervention plays a great role in the provision of information about lifestyle, pregnancy, and delivery. They prevent the potential determinants associated with morbidity and mortality of mothers and children. ANC services not only contribute to pregnant women and their fetus, but also foster a virtuous social and family cohesion and resilience. It was found that more than 80% of pregnant women who attended at least four ANC visits reported showed effectively controlled pregnancy complications. ^33^^,^^36^^,^^37^ ANC services contribute to preparing women for delivery and understanding warning signs during the pregnancy and childbirth. ^38^ It was scientifically found that the ANC interventions attended early become the best opportunity for appropriate screening and medical testing for health problems in which pregnant women are exposed to have during and after pregnancy. ^39^ ANC coverage was an important indicator for reducing the risks to neonatal, infant mortality, maternal mortality and stunting issues to the child. ^37^

Previous studies indicated that there is a substantial influence of ANC on increase of birth weight for the children and their development of a good life characterized by prevention and management of pregnancy-related or concurrent diseases and psychosocial development. ^37^^,^^40^ Maternal health production function explained that early onset of ANC, prenatal care and have a minimum number of ANC and prenatal care visits. ^41^^,^^42^

The rationale of this study was to increase the accessibility to ANC services that were documented to be practiced, but there was no scientific evidence that was conducted to indicate its effect on neither LBW nor contributing determinants to the reduction of children and maternal morbidity and deaths in Rwanda. Through the findings from this research, the investigators indicated how pregnant women achieve the third goal of Sustainable Development Goal (SDG-III) related to the reduction of infant and maternal mortality and morbidity by completing four ANC visits and more. Although it is known that ANC visits are an important intervention that contributes to prediction of the newborn birth weight, little has been conducted on effect of ANC visits on birth weight in developing countries, including Rwanda. This research, therefore, aimed to determine the effect of antenatal care visits on the LBW of children in Rwanda using the secondary data analysis of Rwanda Demographic and Health Survey (RDHS) 2014-2015. We hypothesize that ANC visits result in reducing the high incidence of LBW among newborns of Rwanda.

## Methods

### Data source

The fifth RDHS 2015 was utilized as a nationally representative sample implemented by the National Institute of Statistics of Rwanda (NISR) and Ministry of Health of Rwanda.

### Study technique, participants and settings

The study design was a secondary analysis of cross-sectional survey data from RDHS 2014/2015 that was retrospectively carried out for investigating the effects of antenatal care visits on birth weight of the newborn in Rwanda. The RDHS data collection fieldwork was conducted from November 9, 2014, to April 8, 2015. The data entry, editing, and cleaning was completed by May 15, 2015, and the final survey report was completed in March 2016. A total of 8,004 pregnant women who were to receive antenatal care interventions before delivery were recruited. The interviewed women were of reproductive age (15–49 years). This study was conducted in Rwanda, a small country located in the Central and Eastern Africa bordered by the Republic Democratic of Congo to the West, Uganda to the North, Tanzania to the East and Burundi to the South. This country lies a few degrees south of the equator and is landlocked. Concerning ANC visits, accessibility to ANC services is increasing due to the improvement of the health system and health financing. ^1^ This health system contributes to the achievement of SDG-III those targets reducing morbidity and mortality of mothers and children worldwide specifically in LMICs. The total area of Rwanda is approximately 26,338 km ^2^, the Rwandan population density around 416 people per km ^2^ and the total population is roughly 10.8 million. The majority (43%) of the Rwandan population is aged 15 years or less. Women accounted for about 52.6% of the population, 84% of Rwandans resided in the rural setting, and 71% participant in agricultural activities. ^43^

### Sampling design

RDHS was a national survey conducted to assess the birth weight of newborns. To collect the data of this household-based survey, mothers who had the youngest children, age five years or less, were interviewed to provide data related to birth weight for their children. The data for this survey were collected using a two-stage sampling strategy for enrolling participants. These stages were cluster sampling design and the sampling frame. The sampling frame was composed of the list of the enumerators’ areas (EAs) that covered the entire country. All residents in selected households were eligible to be interviewed. At the first stage of this study, 492 clusters were randomly selected (113 in urban and 379 in rural areas). At the second stage of this study, the systematic sampling technique that focused on selecting the households was applied. Then, a fixed number of 26 households were selected randomly from each cluster and a total of 12,792 households were selected for the final sample for this study.

Additionally, the proportional sampling technique was used in the survey where the sample for each cluster was equal. The study included women aged 15–49 years who were permanent residents of the households or visitors who stayed in the recruited household the night before the survey. For this epidemiological research, this fifth demographic survey presented that although the weight of the newborns at birth is an important predictor of their probability of surviving, current birth weights were not available for the most children. Instead, the mothers were interviewed about the size of their children at birth because this determinant was found to be a proxy for the weight of the newborn. Therefore, 8,004 mothers with 15–49 years of reproductive age were interviewed for reporting the actual weight in kilograms using the written information about birth weight or recalling the weight at birth for their newborns. It was found that 92% of newborns had a birth weight reported while 8% did not.

### Research tools and data collection

RDHS 2014/2015 collected data at the national level using household-based survey data on birth weight retrospectively collected from the mothers. The data collection was completed by trained data collectors who used face-to-face interviews, asking mothers eligible for this study to provide a detailed birth history for children born in the preceding five years. Recruitment included stratified sampling, two stages of cluster sampling designs. The first stage was characterized by selecting the participants from the samples frame constructed from enumeration whereas the second stage involved the systematic sampling of the households. These were listed from each cluster to ensure that an adequate number of the completed individuals were obtained. ^44^ Participants were interviewed based on the measurement of the DHS program. Birth weight was recorded in the RDHS using metric measurement (in kilograms) for all participants from the entire stratum of the country. Data from mothers with stillbirths were excluded from this study.

### Study bias

Bias refers to any tendency or deviation from the truth in study design, data collection, recruiting participants, data analysis, and results interpretation. Generally, bias may occur at any stage of the research. To manage the bias for the data from RDHS, the authors systematically did data cleaning and removed the missing variables. All authors checked several times the selected variables to include in the analysis for minimizing all possible systematic errors that could occur in the study.

### Study variables

**Dependent variable**

The outcome variable of the current study was birth weight of the newborns. As per World Health Organization (WHO) classification, newborns weighing <2,500 grams were categorized as LBW while newborns weigh >2,500 grams were categorized as not having LBW. ^45^

**Explanatory variables**

Based on the literature review and the structure of the RDHS 2014/2015 dataset, the independent variables were found. The main explanatory variable was the number of the antenatal care visits for the pregnant women. Pregnant women who attend none or one ANC visit were considered to have inadequate number of visits, those who attended two or three ANC visits were considered to have intermediate, and those who attended four to seven were taken as adequate number of visits. As recommended by WHO, the pregnant women who attended four ANC visits were considered to have obtained extremely adequate healthcare that effectively contributes to the health of the mother and unborn. ^46^^–^^48^ This study used different covariate variables selected based on the previous epidemiological studies, reviewing the suitable published demographic and epidemiological studies and the available information provided in the demographic health survey (DHS) datasets with the consideration of the potential confounders. Based on the insights from the literature and availability in the datasets, the following variables were included as potential confounders: maternal age (<19 years, 20–34 years and 35–49 years); maternal height (1: <160 cm; 2: ≥160 cm), parity ( *i.e.* birth order), maternal weight (1: <80 kg; 2: 80-89 kg; and 3: ≥90 kg), body mass index (BMI), residence (0: urban; 1: rural); educational attainment (0: illiterate; 1: primary; 2: secondary and higher); household wealth status (0: poor, 1: middle and rich); place of delivery, marital status, maternal occupation, tetanus injection during the pregnancy (0: yes; 1: no), intake iron during the pregnancy (0: yes; 1: no), sex of the child (0: female; 1: male), sex of household head (0: female; 1: male), wanted pregnancy when became pregnant (0: yes; 1: no), blood pressure taken during pregnancy (0: yes; 1: no) provided anti-malaria drugs during pregnancy (0: yes; 1: no), urine sample taken during pregnancy (0: yes; 1: no), received counseling or health education related to nutrients (0: yes; 1: no).

### Statistical analyses

Before analysis, the observations with missing data were dropped. Statistical analysis was performed using descriptive (such as frequency, percentage, standard deviation, and mean) and analytical analyses. In the analytical analysis, bivariate logistic regression and multiple logistic regression models were computed to determine the association between LBW and the explanatory variables. The factors that were significantly associated with LBW at 0.05 or 0.01 in bivariate logistic regression were analyzed using multiple logistic regression models for identifying the association between ANC visits and birth weight when controlling for other covariates, presenting adjusted odds ratio, 95% confidence intervals. Therefore, we adjusted sampling based on the RDHS data that were widely used and consistent data for assessing maternal and child health statistics at the national level using STATA software version 13 (RRID:SCR_012763). ^49^ In this cross-sectional study design, we respected the guidelines outlined in the Strengthening the Reporting of Observational Studies in Epidemiology statement in writing the manuscript. ^50^

### Ethical consideration

Data used were electronically accessed. To get full access, the first registration was completed on the DHS website. The permission to use the 2014/2015 RDHS data was granted by DHS using its website and the prior approval was maintained. In the prior approval, the women of reproductive age who were age 18–49 years provided oral and written informed consent forms to take part in the survey. In the cases on the minor participants (those women aged 15–17 years); the assent form was obtained from them while written informed consent were simultaneously provided by their guardians or parents who were adults.

## Results

### Socio-demographic and economic status of mothers during pregnancy

The results indicated the average birth weight was 3846.1 grams with a standard deviation of 1840.5 grams. The results indicated that the average family size was 5.7 (SD=2). The majority [6012 (76.6%)] weighed 2,500–4,000grams. The majority (61.3%) of pregnant women were aged 35–39 years. For the occupation of the pregnant women, it was found that the majority (86.1%) were involved in agricultural activities (self-employed). About the participants’ religious beliefs, the majority (96.8%) were Christians. Regarding the information about the households, the results indicated that the majority (21.6%) were the poorest. The majority of them (57.9%) were from the families consisting of four to seven family members and 77.3% resided in rural setting. 92% of the pregnant women attended ANC at health centers. Moreover, the prevalence of LBW and macrosomia was 5.8% and 17.6%, respectively, whereas 76.6% of pregnant women delivered newborns who weighed between 2.5 kg and 4 kg. The number of pregnant women who attended four or more ANC visits was 44.1%, those who attended fewer than four ANC was 55.1%, and only 0.8% attended no ANC visits. This indicated that Rwanda did not yet achieve the SDG-III. Besides, the findings showed that during pregnancy, these women are provided different health education and measurement for improving the health of the children including normalizing the birth weight for both mother and their children. Within this period, the results indicated that the majority of the pregnant women (80.5%) were provided at least one tetanus injection before birth, but 19.5% were not. Before pregnancy, the results indicated that the majority was not given any tetanus injection. This means that the tetanus injection is mostly attended by the women when they were pregnant during the ANC visits. The results indicated that more than 70.7% of the pregnant women were provided counseling and education related to nutrients. Indeed, the findings showed that 79.9% of pregnant women were given iron tablet during the pregnant period. The majority (83.4%) of pregnant women were measured blood pressure ( Table 1).

**Table 1.** Distribution of the maternal characteristics in Rwanda, 2015.

Characteristics	Number	Percentage
**Maternal age (years)**		
15-19	120	4
20-34	2260	74.9
35-49	638	21.1
Mean (SD)	36.8(7.4)	
**Maternal weight**		
<80 kg	2621	33.4
80-89 kg	2922	37.2
≥90 kg	2305	29.4
**Marital status**		
Single	324	10.7
Married/cohabiting	2478	82.1
Widowed/separated/divorced	216	7.2
**Maternal education**		
Illiterate	389	12.9
Primary	2139	70.9
Secondary/higher	490	16.2
**Religion**		
Christian	2922	96.8
Muslim	666	2.1
Traditional	213	1.1
**Smoking**		
Yes	63	0.9
No	7319	99.1
**Residence**		
Urban	685	22.7
Rural	2333	77.3
**Household wealth index**		
Poor	236	51.5
Middle/rich	222	48.5
**Family size (persons)**		
Less than 4	282	19.3
4 to 7	843	57.9
More than 7	332	22.8
Mean (SD)	5.7(2)	
**Birth weight**		
<2,500grams	458	5.8
2,500-4,000grams	6012	76.6
>4000grams	1378	17.6
Mean (SD)	3846.1(1840.5)	
**Parity**		
1-2	13	2.2
3-4	138	23.5
≥5	436	74.3
**Injection of tetanus before birth**		
No injection	1161	19.5
Yes	4792	80.5
**Tetanus injection before pregnancy**		
No injection	833	30.4
Yes	1858	67.7
More than 7	52	1.9
**During pregnancy, was given iron tablet**		
No	1198	20.1
Yes	4756	79.9
**Sex of household head**		
Male	729	50
Female	728	50
**Places of ANC**		
Homes	589	2.0
Provincial/district hospital	2166	7.2
Health center	4836	16.1
Privates and other health facilities	262	74.7
**Number ANC visits**		
No ANC and less than 4 ANC	3329	55.9
≥4 ANC	2622	44.1
**Blood pressure taken during pregnancy**		
No	53	16.6
Yes	266	83.4
**Blood sample taken during pregnancy**		
No	9	2.8
Yes	310	97.2
**Heard about health nutrients at Community health workers (CHWs)**		
No	908	42
Yes	1251	58
**Heard about counseling or education on health nutrients**		
No	893	29.3
Yes	2159	70.7

***Notice: SD:** Standard Deviaton*.

In the bivariate logistic regression, we indicated how low birth weight is associated with ANC services, socio-demographic characteristics, maternal influences and behaviors. Therefore, we found that LBW is significantly associated with the ANC visits, BMI, maternal height, maternal weight, residence, place of delivery, sex of child, HWI, maternal parity, maternal, anemia for pregnant women, family size, age, maternal education, provision of tetanus injection during the pregnancy, health education about pregnant complications, provision of counseling about nutrients during pregnancy, provision of malaria drugs, use of health card, and attendance of ANC visits at health center ( Table 2).

**Table 2.** Bivariate logistic regression for analysing the association between the independent variables and LBW.

Characteristics	LBW, n (%)	Odds ratio	95% CI	*p-value*
**Type of residence**				
Urban	75(16.4)	**1**		
Rural	383(83.6)	1.3	1.1-1.7	0.031*
**Body Mass index (BMI)**				
≤18.5 kg/m ^2^	367(80.2)	**1**		
18.5-25.9 kg/m ^2^	60(13.1)	0.4	0.1-1.1	0.02*
≥25 kg/m ^2^	31(6.7)	1.9	1.1-3.3	0.0.3*
**Parity**				
1-2	49(10.7)	**1**		
3-4	338(73.8)	1.9	1.1-3.7	0.003*
≥5	71(15.5)	2.8	1.1-7.2	0.031*
**Maternal height**				
Less than 160 cm	187(40.8)	**1**		
160 cm and more	271(59.2)	2.4	1.3-4.5	0.04*
**Maternal weight**				
<80 kg	200(43.7)	**1**		
80-89 kg	133(29.1)	2.4	1.2-4.8	0.02*
≥90 kg	125(27.2)	2.8	1.3-5.9	0.0009*
**Sex of child at birth**				
Male	203(44.3)	**1**		
Female	255(55.7)	0.8	0.6-0.9	0.014*
**Household Wealth index**				
Poor	236(51.5)	**1**		
Richer/middle	222(48.5)	0.5	0.1-0.7	<0.001**
**Marital status**				
Single	129(28.2)	**1**		
Married/cohabited	263(57.4)	0.2	1.7-2.9	<0.001**
Widowed/separated/divorced	66(14.4)	0.1	0.1-0.2	<0.001**
**Wanted pregnancy when became pregnant**				
Wanted then	290(63.3)	**1**		
Wanted later	114(24.9)	1.8	0.8-1.3	0.673
Wanted no more	54(11.8)	1.1	0.8-1.5	0.405
**Husband’s desire children**				
Both want same	279(61)	**1**		
Husband wants more	49(10.8)	0.8	0.6-1.1	0.211
Husband wants fewer	92(20.1)	1.1	0.8-1.4	0.610
Unaware	37(8.1)	0.99	0.8-1.2	0.956
**Place of delivery**				
Household	16(3.5)	**1**		
Public hospitals	176(38.4)	1.3	0.3-5.8	0.741
Health center	257(56.1)	0.3	0.2-0.6	<0.001**
Privates health facilities	9(2)	0.3	0.2-0.6	<0.001**
**Maternal age**				
15-19	12(2.6)	**1**		
20-34	342(74.7)	2.7	1.5-4.9	<0.001**
35-49	104(22.7)	14.6	7.9-27.1	<0.001**
**Family size**				
Less than 6	297(64.8)	**1**		
6 members and more	161(35.2)	0.5	0.4-0.6	<0.001**
**Smoking for pregnant women**				
Yes	14(3)	**Ref**.		
No	444(97)	0.3	0.1-0.6	0.003*
**Health card**				
No	57(12.5)	**1**		
Yes	401(87.5)	0.3	0.2-0.7	0.003*
**Household head**				
Male	351(76.6)	**1**		
Female	107(23.4)	1.2	0.7-1.02	0.074
**Anemia**				
Not anemic	387(84.4)	**1**		
Anemic	71(15.6)	4.7	2.5-9.2	<0.001**
**Maternal education**				
Illiterate	69(15.1)	**1**		
Post-primary/vocational	335(73.1)	0.5	0.3-1.3	0.105
Secondary and above	54(11.8)	0.3	0.1-0.9	0.036*
**Utilization of Antenatal care services during pregnancy**				
≥ 4 ANC visits	158(34.5)	**1**		
No and less than 4 ANC visit	300(65.5)	1.5	1.2-1.9	<0.001*
**During pregnancy, Immunized with recommended dose of Tetanus and diphtheria**				
No	49(10.7)	**1**		
Yes	409(89.3)	0.3	0.1-0.2	<0.001*
**In pregnancy, provided blood pressure taken**				
No	76(16.6)	1		
Yes	382(83.4)	0.8	0.6-1.1	0.193
**In pregnancy, provided urine sample taken**				
No	198(43.3)	**1**		
Yes	260(56.7)	0.87	0.1-1.1	0.130
**In pregnancy, who provided blood sample taken**				
Yes	13(2.8)	**1**		
No	445(97.2)	1.04	0.5-2.1	0.905
**Told about pregnancy complications**				
Yes	371(81.1)	**1**		
No	87(18.9)	1.1	0.8-1.5	0.0054*
**In pregnancy, consumption of recommended dose of Iron and folic acid**				
No	95(20.8)	**1**		
Yes	363(79.2)	0.4	.12-1.02	0.001**
**During pregnancy, given coartem for malaria treatment**				
No	395(86.3)	**1**		
Yes	63(13.7)	2	1.4-2.8	<0.001**
**During pregnancy, took quinine for malaria**				
No	453(98.8)	**1**		
Yes	5(1.2)	1.1	0.4-3	0.871
**During pregnancy, took other drug for malaria**				
No	449(98.1)	**1**		
Yes	9(1.9)	4.1	1.7-10.2	0.002*
**During pregnancy, provided counseling about nutrients**				
Yes	252(55)	**1**		
No	206(45)	6	3.5-10.7	<0.001**
**Utilization of antenatal care services at health center**				
No	14(3.1)	**1**		
Yes	444(96.9)	0.5	0.3-0.9	0.02*
**During pregnancy, not provided medical drug for malaria taken**				
No	76(16.5)	**1**		
Yes, no drug taken	382(83.5)	0.5	0.4-0.7	<0.001**

**Notice**: (*) Indicates the statistical significance level at 0.05; (**) Estimates the statistical significance level at 0.01; Unmarked p-values were not fond to be the significant risk factors of LBW in Rwanda.

### Multiple logistic regression analyses of the effects of the explanatory variables on birth weight of the newborns in Rwanda

Multiple logistic regression analyses indicated that ANC check-ups are the risk factor of LBW among the pregnant women. Mother attending fewer than four ANC visits are almost three-times more likely to give birth to LBW children [aOR=2.8; 95%CI (1.5–5.4), p=.002] when compared with mothers attending four or more ANC visits. Besides, newborns who were delivered at the health center [aOR=0.3; 95%CI (0.2–0.7), p=.002] and private health institutions [aOR=0.3; 95%CI (0.2–0.6), p<0.001] were less likely to weigh less than 2.5 kg than who were delivered in their households. The findings demonstrated that pregnant women who were not provided Coartem for malaria treatment [aOR=0.6; 95%CI (0.4–0.8), p<0.001] or who received other malaria drugs [aOR=4.1;95CI (1.7–10), p=0.02] were more likely to have LBW newborns. The pregnant women who attended the ANC visits at the health center [aOR=0.5; 95%CI (0.3–0.9), p=0.02] were less likely to have newborns with less than 2.5 kg. For marital status, married and cohabiting pregnant women were 1.86-times at greater risk to have LBW children [aOR=1.9; 95%CI (1.3–2.8), p=0.002] compared with single pregnant women. Mothers who were widowed, divorced and separated from their husbands were more likely to have LBW children compared with the women who were single during pregnancy. The results indicated that the widowed, separated and divorced pregnant women had 1.7-times greater risk of having LBW babies [aOR=1.7; 95%CI (1.1–2.5), p=0.015] than women who were single during pregnancy. Results found that being provided maternal iron and folic acid supplementation during pregnancy [aOR=0.5, 95%CI (0.3–0.9), p=0.015], not being given nutrients during pregnancy [aOR=2.5, 95%CI (2-8.4), p<0.001], being told about maternal complications during pregnancy [aOR=0.7, 95%CI (0.5–1), p=0.05], pregnant women with anemia [aOR=3.5, 95%CI (1.5–9), p<0.001] were positively associated with LBW. Through the number of the pregnant women who smoked during pregnancy, it was found that birth weight is significantly associated with smoking, as pregnant women who did not smoke were less likely [aOR=0.3; 95%CI (0.1–0.6), p=0.002] to deliver LBW babies ( Table 3).

**Table 3.** Multivariate logistic regression for the risk factors associated with low birth weight.

Characteristics	Low birth weight	Adjusted odds ratio		
	Percent		95% CI	*p-value*	
**Type of residence**					
Urban	16.4	**1**			
Rural	83.6	1.1	0.7-1.6	0.008*	
**Maternal education**					
Illiterate	15.1	**1**			
Primary/vocational	73.1	1.01	0.7-1.5	<0.001**	
Secondary/higher	11.8	0.99	0.6-1.8	<0.001**	
**Maternal age**					
15-19	2.6	**1**			
20-34	74.7	0.8	0.4-1.5	0.428	
35-49	22.7	0.8	0.4-1.5	0.467	
**Provided Tetanus injection during pregnancy**					
Yes	89.3	**1**			
No	10.7	1.3	0.8-2.1	0.037*	
**Body Mass index (BMI)**					
≤18.5 kg/m ^2^	80.2	**1**			
18.5-25.9kg/m ^2^	13.1	0.9	0.4-2.3	0.008*	
≥25kg/m ^2^	6.7	0.5	0.2-1.1	0.005*	
**Maternal height**					
Less than 160cm	40.8	**1**			
160cm and more	59.2	0.5	0.2-1.2	0.11	
**Smoking during pregnancy**					
Yes	3	**1**			
No	97	0.3	0.1-0.6	0.002*	
**Maternal weight**					
<80kg	43.7	**1**			
80-89kg	29.1	0.4	0.1-1.1	0.003*	
≥90kg	27.2	1.7	0.8-3.5	0.03*	
**Utilization of Antenatal care services**					
4 ANC and more	34.5	**1**			
No and less than ANC	65.5	2.8	1.5-5.4	0.002*	
**Parity for pregnant women**					
1-2	10.7	**1**			
3-4	73.8	0.5	0.2-1.1	0.2	
≥5	15.5	0.7	0.4-1.4	0.041	
**Family size**					
Less than 6	64.8	**1**			
6 members and more	35.2	1.02	0.8-1.4	0.918	
**Household Wealth Index**					
Poor	51.5	**1**			
Middle/rich	48.5	0.9	0.6-1.4	0.728	
**Anemia**					
Not anemic	84.4	**1**			
Anemic	15.6	3.5	1.5-8.6	<.001*	
**Marital status**					
Single	15.5	**1**			
Married/cohabited	75.5	1.9	1.3-2.8	0.002*	
Widowed/separated/divorced	9.0	1.6	1.1-2.5	0.015*	
**During pregnancy, not given coartem for malaria**					
No	86.3	**1**			
Yes	13.7	0.6	0.4-0.8	<0.001*	
**During pregnancy, no drug for malaria taken**					
No	16.5	**1**			
Yes, no drug taken	83.5	0.3	0.1-0.8	0.018*	
**During pregnancy, provided counseling about nutrients**					
Yes	55	**1**			
No	45	2.5	2-8.4	<0.001*	
**Place of delivery**					
Household	3.5	**1**			
Public hospitals	38.4	1.03	0.2-4.8	0.969	
Health center	56.1	0.3	0.2-0.7	0.002*	
Privates	2	0.3	0.2-0.6	<0.001*	
**Sex of child at birth**					
Male	44.3	1			
Female	55.7	0.8	0.62-1.16	0.293	
**Provided tetanus injection during pregnancy**					
No	15.2	**1**			
Yes	84.8	0.3	0.8-2	0.337	
**Antenatal care visits at health center**					
No	3.2	**1**			
Yes	96.8	1.9	0.9-4.1	0.109	
**In pregnancy, consumption of recommended dose of Iron and folic acid**					
No	79.2	**1**			
Yes	20.8	0.5	0.3-0.9	0.015*	
**Told about complications during pregnancy**					
Yes	81.1	**1**			
No	18.9	3.3	1.5-6.6	0.003*	
**Health care access**					
No	12.5	**1**			
Yes	87.5	0.7	0.5-1	0.05*	
**Household head**					
Male	76.6	**1**	1		
Female	23.4	1.1	0.8-1.6	0.519	

**Notice**: * Indicates statistical significance at p-value <0.05; ** Indicates significance at 0.01, Unmarked p-values were not fond to be the significant risk factors of LBW in Rwanda.

## Discussion

This study revealed that LBW remains a public health burden at the national level and it remains higher in Rwanda. The results found that the prevalence of LBW and macrosomia was 5.8% and 17.6%, respectively; however, the newborns weighing more than 2.5kg were 94.2%. These results were coincided with the previous studies that documented an improvement of ANC services contributing to birth weight of the newborns. ^34^ Similarly, the prevalence of LBW is low compared with other counties in the same region such as Zambia, Uganda and Nigeria. ^51^^–^^53^ It is also less than the prevalence of some countries in Asia such as in China where the accessibility to ANC visits remains challenging. ^54^ The present prevalence of LBW is less than the previous document which was 7.1% and low access of four recommended ANC visits was 35.4%. ^55^ There is a significant reduction of the prevalence of LMICs compared with the prevalence (15.9%) found in developing countries. ^6^ Among the women with the newborns who weigh less than 2.5kg, only 44.1% of pregnant women attended four recommended ANC visits. Within this study, a strong correlation between ANC and LBW was discovered and the results of inadequate ANC visit for pregnant women. However, almost all pregnant women (99%) attended ANC visits and only 44.1% obtained 4 recommended ANC packages, there is a need to increase attendance of ANC visits for reducing the number of newborns with LBW. These results corroborated preceding studies. ^54^^,^^56^

We observed that pregnant women who attended four and more ANC visits had less risk of having LBW children than those who attended fewer than four recommended ANC visits. This finding is consistent with previous data that indicated that the more pregnant women attend ANC visits, especially more than four, the more their children do not have LBW. ^57^^,^^58^ Pregnant women who attended fewer than four ANC check-ups had 2.8-times greater odds of LBW than those who attended four and more ANC visits. These findings are relevant with the previous studies that indicated that ANC visits are significant maternal factors contributing to birth weight babies, since it normalizes the birth weight of the child and their mother due to the prevention, intervention and health education that are effectively provided in each visit. ^54^^,^^59^^,^^60^ They are also relevant to prior studies conducted in central African counties that indicated that the attendance of ANC visits and being provided the recommended packages for pregnant women reduce the risk for delivering LBW outcome. ^27^^,^^61^^–^^63^ Results revealed that the pregnant women who were not provided Coartem for malaria treatment were 0.6-times likely to have LBW children compared with the pregnant women who were provided Coartem for malaria treatment. ^57^^,^^64^ It was found that the pregnant women who received no drug for malaria treatment had 0.3-times greater risk of having LBW children than the women who received the drugs for malaria. ^21^ These results were similar to the previous studies that documented that children born to educated pregnant women and pregnant women with high wealth index who attend are less likely to weigh less than 2.5kg than other pregnant women. ^32^^,^^65^ Prominently, in this research, the results are robust to adjustment for the socio-demographic characteristics of the recruited pregnant women. They revealed that marital status increased the odds of LBW. It was found that pregnant women who were married or living with the cohabitants were 1.9-times likely to have LBW compared with pregnant women who were single. They also revealed that the pregnant women who divorced or separated had 1.7-greater risks to have newborns who weigh less than 2.5 kg. These results challenged the previous studies that indicated that pregnant women who were single had a greater risk of having LBW children than others. ^52^^,^^66^^,^^67^

Newborns whose mothers were had normal BMI (18.5–24.9 kg/m ^2^) and mothers who were overweight (or obese;) were less likely to have new-born with LBW when compared to those mothers who were underweight. This result is consistent with the earlier research carried out in Uganda. ^68^ Newborns of women who smoke during pregnancy had low birth weight when compared with babies whose mothers did not smoke during pregnancies. Our findings are in congruence with the studies conducted in Tanzania. ^69^ Mothers from rural areas had 1.1 times greater risks of having an LBW newborn than those residing in urban settings. Mothers with secondary and higher education were less likely to have an LBW newborn than those who were illiterate. Our results concur with the earlier studies that established that the residence of pregnant women, HWI, family size, maternal age, utilization of tetanus injections and iron tablets consumption are factors of LBW. ^42^^,^^69^

Maternal anemia was identified as a risk factor, where we found that pregnant women with anemia had 3.5- times greater odds of delivering a newborn who weighed less than 2.5 kg than those who are not anemic. This result is in line with the prior studies conducted in Nigeria. ^52^ The mothers who were not provided health counseling and education about nutrients were 2.5-times more likely to have LBW newborns than those who received nutritional counseling and education during ANC visits. The mothers who were not informed about maternal complications had 3.3-times greater risks of having newborns who weighed less than 2.5 kg than those who received information about health complications during pregnancy. Mothers with a weight at delivery greater than 80 kg were more likely to deliver newborn with LBW than the mothers who weighed less than 80 kg. This result is not in line with the previous studies carried out in Ethiopia. ^70^ The findings of this study did not reveal a significant association between maternal age, parity, HWI, sex of child, family size and LBW, however, the prior studies conducted in sub-Saharan African countries confirmed a significant association between maternal age, parity, sex of child, HWI, and LBW. ^52^^,^^70^

## Strengths and limitations

The present research has numerous prominent strengths. The first strength is that the RDHS 2014/2015 used the validated and standardized tools for interviewing the eligible participants. Second, the research data about birth weight that we used were obviously verified through records and recall bias was prevented. However, several limitations also warrant discussion. First, it was limited to a study design that did not actually explore for modifiable factors of ANC visits, such as HIV status of pregnant women, maternal gestational age, prenatal depression, receiving the supplementary vitamins (like vitamin A) during the pregnant period, maternal weight gain during pregnancy, eclampsia, gestational diabetes, high blood glucose level during pregnancy, antiretroviral (ART) for HIV-positive women and reducing transmission of HIV from pregnant mother to child. Second, birth weight data was available for only babies whose weight was measured at birth. This limited us for estimating the real prevalence. Third, the selection bias resulted in the underestimation of the association between ANC and birth weight. This was because not all babies’ weight was measured. The other limitation was that the qualitative aspects data from the participants was not included for exploring some factors and to triangulate the findings of the quantitative methods used. Additionally, as the methods used for measuring birth weight were not validated by the survey team, the misclassification could occur in this research. As we do not know the exact timing of the birth weight measurement, this causes misclassification.

## Conclusion

In conclusion, this study found several risk factors of LBW in Rwanda that remains a national public health concern. However, ANC services for pregnant women are a fundamental intervention for reducing LBW. This integral part of primary health care, ANC services, is the population-based intervention that reduces the risk to deliver the newborn with LBW. Therefore, there is a need of a multifaceted approach for addressing the factors of LBW through reinforcing ANC coverage and quality of ANC services for pregnant women. Information on anthropometric measurement and increased awareness of the importance of regular ANC visits is also desirable. Attending four or more ANC visits is a step toward improving maternal health by providing maximum required packages and this strategy reduces the risk to LBW. The policy makers and researchers should prioritize maternal education and families with low socio-economic status for preventing LBW in Rwanda.

We recommend further research be conducted on the risk factors of macrosomia in Rwanda. Additionally, further study to explore the barriers to access ANC visits effectively for pregnant women from the rural settings is recommended. In the meantime, pregnant women in Rwanda are required to attend ANC visits appropriately and regularly for benefiting from the required packages that increase the probability to attenuate the LBW among the newborns. We recommend to measure birth weight immediately after birth for reducing the recall bias and misclassification.

## Data availability

### Underlying data

Underlying data for ‘Regular antenatal care visits were associated with low risk of low birth weight among newborn in Rwanda: Evidence from the 2014/2015 Rwanda Demographic Health Survey (RDHS) Data was owned by the DHS program that can be obtained from https://dhsprogram.com/methodology/survey/survey-display-468.cfm.

The electronic data is available from the DHS program under its terms of use. Before downloading the data, the main author of this study registered as DHS user for reasons laid out on the DHS program website and dataset access was only granted for legitimate purpose of this research.

## Authors’ contributions

EB conceptualized the study, did study design, formal analysis, wrote the protocol, requested the permission from the DHS website, drafted the manuscript and coordinated the study. SH contributed to data analysis, data curation and methodology. DR substantially contributed to the conceptualization. He also wrote the original draft preparation. He revised and reviewed the manuscript, and searched the relevant journal to which the study is submitted. HE reviewed and edited the study. All investigators approved the final manuscript. They also agreed to take responsibility and be accountable for the content of the manuscript. All of them agreed on all versions of the manuscript before submitting it in the international journal. They also agreed on the final version accepted for publication.

## Ethics and consent to participate

The present study was exempt from searching the ethical approval because the RDHS 2014/15 obtained the ethical approval from the Institutional Review Board (IRB) of the Rwanda National Ethics Committee (RNEC).
