# Regular antenatal care visits were associated with low risk of low birth weight among newborns in Rwanda: Evidence from the 2014/2015 Rwanda Demographic Health Survey (RDHS) Data

**DOI:** 10.12688/f1000research.51969.2

**Published:** 2022-11-03

**Authors:** Emmanuel Biracyaza, Samuel Habimana, Donat Rusengamihigo, Heather Evans

**Affiliations:** 1 Department of Community Health, University of Rwanda, Kigali, Rwanda; 2 Sociotherapy Program, Prison Fellowship Rwanda (PFR), Kigali, Rwanda; 3 Executive Director, Rwanda Resilience and Grounding Organization (RRGO), Kigali, Rwanda; 4 Department of Clinical Psychology, University of Picardy Jules Verne, Amiens, France; 5 Evans Counseling Services, Coopersburg, USA

**Keywords:** Antenatal care; Birth weight; newborn; Maternal factors; Sustainable Development Goals; Pregnancy

## Abstract

**Background:** Low birth weight (LBW) remains the global unfinished agenda in most countries of the world especially in low- and middle-income countries. LBW subsequently has harmful effects on the lifestyle, psychosocial and physiological development of the child. Although it is known that antenatal care (ANC) visits are important interventions contributing to prediction of newborn birth weight, little has been conducted on effect of ANC visits on birth weight in Rwanda. This study aimed at determining the association between regular ANC visits and risk of LBW among newborns in Rwanda.

**Methods: **A cross-sectional study design was conducted to analyse the effects of ANC on LBW using the 2014/2015 Rwanda Demographic Health Survey. Associations of socio-demographic, socio-economic, and individual factors of the mother with LBW newborns were performed using bivariate and multiple logistic regression analyses.

**Results**: Prevalence s of LBW and macrosomia were 5.8% and 17.6%, respectively. Newborns delivered from mothers attending fewer than four ANC visits were at almost three-times greater risk of having LBW [aOR=2.8; 95%CI (1.5–5.4), p=0.002] compared to those whose mothers attending four or more ANC visits. Residing in a rural area for pregnant women was significantly associated with LBW [aOR=1.1; 95%CI (0.7–1.6), p=0.008]. Maternal characteristics, such as anemia, predicted an increase in LBW [aOR=3.5; 95%CI (1.5–5.4),p<0.001]. Those who received no nutritional counseling [aOR=2.5; 95%CI (2–8.5), p<0.001] and who were not told about maternal complications [aOR=3.3; 95%CI (1.5–6.6), p=0.003] were more prone to deliver newborns with LBW than those who received them. Pregnant women who received iron and folic acid were less likely to have LBW newborns [aOR=0.5; 95%CI (0.3–0.9), p=0.015].

**Conclusion**: ANC visits significantly contributed to reducing the incidence of LBW. This study underscores the need for early, comprehensive, and high-quality ANC services to prevent LBW in Rwanda.

## Introduction

Antenatal care (ANC) globally is an important health strategy that has been considered as an essential intervention to contribute to the health of newborns and their mothers. This health intervention reduces low birth weight (LBW) which is a foremost public health burden that weakens the development of families and nations due to its harmful effects on quality of life ^1^. LBW is defined when a baby weighs less than 2.5 kilograms at birth. This health burden particularly, in low- and middle-income countries (LMICs) may cause multiple effects, such as birth asphyxia, amniotic fluid aspiration, hypoglycemia and hyponatremia ^2– 4^. The estimate of 15.5% to 15.9% of LBW worldwide was reported ^5, 6^ and the high prevalence (95.6%) of LBW newborns occurred in LMICs ^7, 8^. ANC visits were found to play a great role in the health promotion of mothers and children before, during and after delivery. It specifically contributes to the reduction of morbidity and mortality of mothers and children ^9, 10^. Epidemiological studies have shown that the children weighing less than 2.5 kilograms are nearly 20-times more likely to die than those who weigh 2.5 kilograms and more ^11^. Other studies have stated that newborns with LBW were 5–10-times more likely to die than those who weighed 2.5 kilograms and more ^2, 3^. Prior studies have indicated that the newborn may develop macrosomia, defined as birth weight more than 4 kilograms. These newborns are considered to have an excessive birth weight. Its prevalence varies globally between 3 and 15% depending on the region, due to various factors where it remains higher in the developing world ^12, 13^.

Prior studies indicated that maternal and environmental factors are major factors with regard to LBW ^14, 15^. The number of ANC visits attended by the pregnant women and the amount of recommended packages of the interventions provided during ANC are the main factors contributing to LBW ^14, 16^. A low socio-economic status and limited access to qualified health services related to pregnancy are risk factors for LBW ^16^. Multiple studies established that accessibility to poor food consumption behaviors, problems of health status for pregnant women, low caloric intake, hypertension, urinary infections, smoking behaviors, genital infections and psychological distress were found to be risk factors of LBW ^4, 17, 18^. Earlier studies conveyed that consumption of iron, birth order, and calcium supplementation reduces the risk to develop LBW ^18– 20^.

ANC refers to the routine health management of presumed healthy pregnant women without the symptomatology or screening so as to diagnose and detect the diseases or complicating symptoms ^16, 21, 22^. The World Health Organization (WHO) recommended pregnant women attend a minimum of four ANC visits for obtaining the basic health education and interventions that promote health of babies. The more they attend ANC services, the more they receive the maximum health package that plays a crucial role in their health and the health of their newborn. Prior studies have documented that two-thirds of all pregnant women worldwide attended at least one ANC service during pregnancy. They also documented that ANC is the significant predictor of birth weight where attending the required ANC increases the probability of achieving full life-saving potential and having a newborn with more than 2,500 grams ^23– 25^. In ANC visits, pregnant women are offered health education, such as adequate nutrients to be consumed during and after delivery, vitamin intake, proper vaccination, changing risk behaviors such as smoking, and place of delivery ^26, 27^. The package provided to pregnant women consists of many interventions like the identification and management of the obstetric complications including pre-eclampsia, tetanus toxoid immunization, de-worming, iron and folic acid. The fundamental rudiments of a focused approach to ANC are surveillance of health issues among pregnant woman and their babies, management of complications occurring in the pregnancy period, such as hypertension, treatment of underlying or concurrent illness, screening for health conditions and diseases, mental health problems and intra-partner violence or domestic violence ^24, 27, 28^. ANC visits are obviously the opportunities for promoting the use of skilled attendance at birth, injury prevention, adherence support for preventive interventions, healthy lifestyle safety, and healthy behaviors such as breastfeeding, early postnatal, and planning for the optimal pregnancy spacing ^23, 29, 30^. Pregnant women are provided supplementary nutrients for enhancing the baby’s and mother’s health, screening for genetic and congenital disorders, and offered folic acid supplementation to reduce the risk of neural tube defects ^1, 8, 26, 31, 32^.

Earlier researchers documented that through ANC services, pregnant women and their newborns develop physiological and psychosocial wellbeing. It also reduces risk to have a LBW newborn. ANC visits contributed to development of healthy behaviors and compromise of the emergency preparedness plan to intensify the maternal awareness, improving the newborn needs and self-care ^27^. Other studies indicated that ANC visits contribute to weight gain and weight regulation of women. They confirmed that ANC services increase the weight of pregnant women and contribute to the growth of the fetal and maternal tissues and fluids ^21^. This health intervention plays a great role in the provision of information about lifestyle, pregnancy, and delivery. They prevent the potential determinants associated with morbidity and mortality of mothers and children. ANC services not only contribute to pregnant women and their fetus, but also foster a virtuous social and family cohesion and resilience. It was found that more than 80% of pregnant women who attended at least four ANC visits reported showed effectively controlled pregnancy complications ^31, 33, 34^. ANC services contribute to preparing women for delivery and understanding warning signs during the pregnancy and childbirth ^35^. It was scientifically found that the ANC interventions attended early become the best opportunity for appropriate screening and medical testing for health problems in which pregnant women are exposed to have during and after pregnancy ^36^. ANC coverage was an important indicator for reducing the risks to neonatal, infant mortality, maternal mortality and stunting issues to the child ^34^.

Previous studies indicated that there is a substantial influence of ANC on increase of birth weight for the children and their development of a good life characterized by prevention and management of pregnancy-related or concurrent diseases and psychosocial development ^34, 37^. Maternal health production function explained that early onset of ANC, prenatal care and have a minimum number of ANC and prenatal care visits ^38, 39^.

The rationale of this study was to increase the accessibility to ANC services that were documented to be practiced, but there was no scientific evidence that was conducted to indicate its effect on neither LBW nor contributing determinants to the reduction of children and maternal morbidity and deaths in Rwanda. Through the findings from this research, the investigators indicated how pregnant women achieve the third goal of Sustainable Development Goal (SDG-III) related to the reduction of infant and maternal mortality and morbidity by completing four ANC visits and more. Although it is known that ANC visits are an important intervention that contributes to prediction of the newborn birth weight, little has been conducted on effect of ANC visits on birth weight in developing countries, including Rwanda. This research, therefore, aimed to determine the effect of antenatal care visits on the LBW of children in Rwanda using the secondary data analysis of Rwanda Demographic and Health Survey (RDHS) 2014–2015. We hypothesize that ANC visits result in reducing the high incidence of LBW among newborns of Rwanda.

## Methods

### Data source

The fifth RDHS 2015 was utilized as a nationally representative sample implemented by the National Institute of Statistics of Rwanda (NISR) and Ministry of Health of Rwanda.

### Study technique, participants and settings

The study design was a secondary analysis of cross-sectional survey data from RDHS 2014/2015 that was retrospectively carried out for investigating the effects of antenatal care visits on birth weight of the newborn in Rwanda. The RDHS data collection fieldwork was conducted from November 9, 2014, to April 8, 2015. The data entry, editing, and cleaning was completed by May 15, 2015, and the final survey report was completed in March 2016. A total of 8,004 pregnant women who were to receive antenatal care interventions before delivery were recruited. The interviewed women were of reproductive age (15–49 years). This study was conducted in Rwanda, a small country located in the Central and Eastern Africa bordered by the Republic Democratic of Congo to the West, Uganda to the North, Tanzania to the East and Burundi to the South. This country lies a few degrees south of the equator and is landlocked. Concerning ANC visits, accessibility to ANC services is increasing due to the improvement of the health system and health financing ^1^. This health system contributes to the achievement of SDG-III those targets reducing morbidity and mortality of mothers and children worldwide specifically in LMICs. The total area of Rwanda is approximately 26,338 km ^2^, the Rwandan population density around 416 people per km ^2^ and the total population is roughly 10.8 million. The majority (43%) of the Rwandan population is aged 15 years or less. Women accounted for about 52.6% of the population, 84% of Rwandans resided in the rural setting, and 71% participant in agricultural activities ^40^.

### Sampling design

RDHS was a national survey conducted to assess the birth weight of newborns. To collect the data of this household-based survey, mothers who had the youngest children, age five years or less, were interviewed to provide data related to birth weight for their children. The data for this survey were collected using a two-stage sampling strategy for enrolling participants. These stages were cluster sampling design and the sampling frame. The sampling frame was composed of the list of the enumerators’ areas (EAs) that covered the entire country. All residents in selected households were eligible to be interviewed. At the first stage of this study, 492 clusters were randomly selected (113 in urban and 379 in rural areas). At the second stage of this study, the systematic sampling technique that focused on selecting the households was applied. Then, a fixed number of 26 households were selected randomly from each cluster and a total of 12,792 households were selected for the final sample for this study.

Additionally, the proportional sampling technique was used in the survey where the sample for each cluster was equal. The study included women aged 15–49 years who were permanent residents of the households or visitors who stayed in the recruited household the night before the survey. Instead, the mothers were interviewed about the size of their children at birth because this determinant was found to be a proxy for the weight of the newborn. Therefore, 8,004 mothers with 15–49 years of reproductive age were interviewed for reporting the actual weight in kilograms using the written information about birth weight or recalling the weight at birth for their newborns. But our study inclucted 7381 women (92%) whose their newborns were measued weight. Therefore, 8% of the women whose newborns were not measured weight at birth were not enrolled in this study. Futher, all records on birth weight, number of ANC visits and BMI were available in the RDHS.

### Research tools and data collection

RDHS 2014/2015 collected data at the national level using household-based survey data on birth weight retrospectively collected from the mothers. The data collection was completed by trained data collectors who used face-to-face interviews, asking mothers eligible for this study to provide a detailed birth history for children born in the preceding five years. Recruitment included stratified sampling, two stages of cluster sampling design. The first stage was characterized by selecting the participants from the samples frame constructed from enumeration whereas the second stage involved the systematic sampling of the households. These were listed from each cluster to ensure that an adequate number of the completed individuals were obtained ^41^. Participants were interviewed based on the measurement of the DHS program. Birth weight was recorded in the RDHS using metric measurement (in kilograms) for all participants from the entire stratum of the country. Data from mothers with stillbirths were excluded from this study.

### Study bias

Bias refers to any tendency or deviation from the truth in study design, data collection, recruiting participants, data analysis, and results interpretation. Generally, bias may occur at any stage of the research. To manage the bias for the data from RDHS, the authors systematically did data cleaning and removed the missing variables. All authors checked several times the selected variables to include in the analysis for minimizing all possible systematic errors that could occur in the study.

### Study variables

***Dependent variable*.** The outcome variable of the current study was birth weight of the newborns. As per World Health Organization (WHO) classification, newborns weighing <2,500 grams were categorized as LBW while newborns weigh >2,500 grams were categorized as not having LBW ^42^.

***Independent variables*.** Based on the literature review and the structure of the RDHS 2014/2015 dataset, the independent variables were found. The main independent variable was the number of the ANC visits for the pregnant women. Although we expected to use a cut-off of 8 ANC visits as recommended by the WHO, a low prevalence (1%) of utilising performing 8 recommended ANC visits did not allow to use this appropriate recoommendation. Thus, we considered a cut of 4 ANC visits and considered that the pregnant women who attended less than 4 ANC visits and those who attended 4 and above ANC visits were inadequate and adequate respectively. As recommended by WHO in 2010, the pregnant women who attended 4 ANC visits were considered to have obtained extremely adequate healthcare that effectively contributes to the health of the mother and unborn ^43, 44^. This study used different covariate variables selected based on the previous epidemiological studies, reviewing the suitable published studies and the available information provided in the demographic health survey (DHS) datasets with the consideration of the potential confounders. Based on the insights from the literature and availability in the datasets, such factors are socio-demographic data such as maternal maternal age, residence, educational attainment, household wealth status, place of delivery, marital status, maternal occupation, gender of the child, sex of household head. In additional to independent variables, we also had linear variables that compromise the variables such as body mass index (BMI), anemia and nutritional supplements including tetanus injection during the pregnancy, iron folic supplementation, and nutritional counseling during pregnancy.

### Statistical analyses

Before analysis, the observations with missing data were dropped. Statistical analysis was performed using descriptive (such as frequency, percentage) and analytical analyses. In the analytical analysis, bivariate logistic regression analyeses were performed and all significant explanatory variables at p<0.25 were included in the multivariate logistic regression models based on the odd ratios to determine the associated factors of LBW, presenting adjusted odd ratios with a consideration of 95% for the confidence intervals. Further, all determinants in the multivariate logistic regression models were assessed for collinearity, which was considered present if the study variables had a variance inflation factor (VIF) higher than 3. Therefore, we adjusted sampling based on the RDHS data that were widely used and consistent data for assessing maternal and child health statistics at the national level using STATA software version 13 (RRID:SCR_012763) ^45^. In this cross-sectional study design, we respected the guidelines outlined in the Strengthening the Reporting of Observational Studies in Epidemiology statement in writing the manuscript ^46^.

### Ethical consideration

Data used were electronically accessed. To get full access, the first registration was completed on the DHS website. The permission to use the 2014/2015 RDHS data was granted by DHS using its website and the prior approval was maintained. In the prior approval, the women of reproductive age who were age 18–49 years provided oral and written informed consent forms to take part in the survey. In the cases on the minor participants (those women aged 15–17 years); the assent form was obtained from them while written informed consent were simultaneously provided by their guardians or parents who were adults.

## Results

### Socio-demographic and economic status of mothers during pregnancy

Our of 7381 women, the majority (61.3%) of pregnant women were aged 35–39 years and 82.1% were married or lived with the cohabitants. Our results showed that the majority (46.1%) were Catholics and 55.2% presented normal BMI (18.5–25.9 kg/m ^2^). Regarding behaviors, 0.9% of them were smokers. About 73.1% studies primary schools and majority of them (82.3%) were from rural areas. Concerning nutritional status, 80.5% received tetanus injection, 79.9% received iron and folic acid supplementations, however, 24.1% had anaemia. Further, a majority (66.3% experienced ≥37 weeks, 64.1% were from the families hed by the females and 55.9% delivered females. Indeed, 89% of the women were informed about pregancy commplications and 70.7% provided nutritional counseling during the pregnancy ( Table 1).

**Table 1.** Descriptive analysis for socio-demographic, nutritional, maternal and economic factors (N=7381).

Variables	Number	Percent
**Maternal age (years)**		
15–19	529	4
20–34	5664	74.9
35–49	1188	21.1
**Sex of newbors**		
Males	3254	44.1
Females	4127	55.9
**Marital status**		
Single	790	10.7
Married/cohabiting	6059	82.1
Widowed/separated/divorced	532	7.2
**Maternal education**		
Illiterate	1002	13.6
Primary	5397	73.1
Secondary/higher	982	13.3
**Religion**		
Protestants	3183	43.1
Catholics	3402	46.1
Adventists	513	7
Others	283	3.8
**Smoking**		
Smokers	63	0.9
Non-smokers	7318	99.1
**Residence**		
Urban	1307	17.7
Rural	6074	82.3
**Household wealth index**		
Poor	4178	56.6
Middle/rich	3203	43.4
**Family size (persons)**		
Less than 4	1425	19.3
4 to 7	4273	57.9
More than 7	1683	22.8
**Birth weight**		
<2,500grams (LBW)	436	5.8
2,500–4,000grams (normal)	5646	76.6
>4000grams (macrosomia)	1299	17.6
**Parity**		
1–2	163	2.2
3–4	1734	23.5
≥5	5484	74.3
**Body Mass index (BMI)**		
≤18.5 kg/m ^2^	2293	31.1
18.5–25.9 kg/m ^2^	4076	55.2
≥25 kg/m ^2^	1012	13.7
**Gestational age**		
<37 weeks	2485	33.7
≥37 weeks	4896	66.3
**Received tetanus injections**		
No	1439	19.5
Yes	5941	80.5
**During pregnancy, was given iron tablet**		
No	1484	20.1
Yes	5897	79.9
**Sex of household head**		
Male	2651	35.9
Female	4730	64.1
**Places of ANC**		
Homes	589	8
Provincial/district hospital	2166	26.3
Health center	4363	59.1
Privates and other health facilities	263	3.6
**Number ANC visits**		
Attended 4 or less ANC visits	4133	56
Attended ≥4 ANC visits	3248	44
**Heard about nutrients from CHWs**		
No	3100	42
Yes	4281	58
**Desired children**		
Both want same	4378	59.3
Husband wants more	1349	18.3
Husband wants fewer	1281	17.3
Unaware	373	5
**Maternal anemia**		
No anemic	5599	75.9
Anemic	1982	24.1
**Told about pregnancy complications**		
No	797	10.8
Yes	6584	89.2
**Nutritional counseling during** **pregnancy**		
No	2163	29.3
Yes	5218	70.7

In the bivariate logistic regression, we indicated how low birth weight is associated with ANC services, socio-demographic characteristics, maternal influences and behaviors. Therefore, we found that LBW was significantly associated with the ANC visits, BMI, residence, place of delivery, sex of child, HWI, maternal parity, maternal, anemia for pregnant women, family size, age, maternal education, provision of tetanus injection during the pregnancy, health education about pregnant complications and provided of nutritional counseling during pregnancy ( Table 2).

**Table 2.** Bivariate logistic regression for analysing the association between the independent variables and LBW.

Characteristics	LBW, n (%)	Odds ratio	95% CI	*p-value*
**Type of residence**				
Urban	75(16.4)	**1**		
Rural	383(83.6)	1.3	1.1-1.7	0.031 *
**Body Mass index (BMI)**				
≤18.5 kg/m ^2^	367(80.2)	**1**		
18.5–25.9 kg/m ^2^	60(13.1)	0.4	0.1-1.1	0.02 *
≥25 kg/m ^2^	31(6.7)	0.83	0.73-0.94	0.03 *
**Parity**				
1-2	49(10.7)	**1**		
3-4	338(73.8)	1.9	1.1-3.7	0.030 *
≥5	71(15.5)	2.8	1.1-7.2	0.041 *
**Sex of newborn**				
Male	203(44.3)	**1**		
Female	255(55.7)	0.8	0.6-0.9	0.014 *
**Household Wealth index**				
Poor	236(51.5)	**1**		
Richer/middle	222(48.5)	0.5	0.1-0.7	<0.001 **
**Marital status**				
Single	129(28.2)	**1**		
Married/cohabited	263(57.4)	0.2	1.7-2.9	<0.001 **
Widowed/separated/divorced	66(14.4)	0.1	0.1-0.2	<0.001 **
**Wanted pregnancy when became pregnant**				
Wanted then	290(63.3)	**1**		
Wanted later	114(24.9)	1.8	0.8-1.3	0.673
Wanted no more	54(11.8)	1.1	0.8-1.5	0.405
**Husband’s desire children**				
Both want same	279(61)	**1**		
Husband wants more	49(10.8)	0.8	0.6-1.1	0.211
Husband wants fewer	92(20.1)	1.1	0.8-1.4	0.610
Unaware	37(8.1)	0.9	0.8-1.2	0.956
**Place of delivery**				
Homes	16(3.5)	**1**		
Provincial/district hospital	176(38.4)	1.3	0.3-5.8	0.071
Health center	257(56.1)	0.6	0.1-3.2	0.061
Privates and other health facilities	9(2)	0.2	0.1-0.4	0.051
**Gestational age**				
<37 weeks	187 (40.8)	1		
≥37 weeks	271(59.2)	0.7	0.6-0.8	<0.01 **
**Maternal age**				
15–19	12(2.6)	**1**		
20–34	342(74.7)	2.7	1.5-4.9	<0.001 **
35–49	104(22.7)	14.6	7.9-27.1	<0.001 **
**Family size**				
Less than 6	297(64.8)	**1**		
6 members and more	161(35.2)	0.5	0.4-0.6	<0.001 **
**Smoking for pregnant women**				
No smokers	395(86.2)	**1**		
Smokers	63(13.8)	1.6	1.2-2.1	.02 *
**Household head**				
Male	213(46.6)	**1**		
Female	245(53.4)	1.2	0.7-1.02	0.074
**Anemia**				
Not anemic	201(43.9)	**1**		
Anemic	257(56.1)	4.7	2.5-9.2	<0.001 **
**Maternal education**				
Illiterate	69(15.1)	**1**		
Post-primary/vocational	335(73.1)	0.5	0.3-1.3	0.105
Secondary and above	54(11.8)	0.3	0.1-0.9	0.036 *
**Utilization of Antenatal care services during pregnancy**				
≥ 4 ANC visits	158(34.5)	**1**		
Less than 4 ANC visit	300(65.5)	1.5	1.2-1.9	<0.001 *
**Taken Tetanus and diphtheria pregnancy**				
No	49(10.7)	**1**		
Yes	409(89.3)	0.3	0.1-0.2	<0.001 *
**Told about pregnancy complications**				
No	87(18.9)	1		
Yes	371(81.1)	0.9	0.4-2.7	.001 **
**Iron and folic acid during pregnancy**				
No	95(20.8)	**1**		
Yes	363(79.2)	0.4	0.12-1.02	0.001 **
**Provided nutritional counseling during pregnancy**				
Provided	252(55)	**1**		
Not provided	206(45)	6	3.5-10.7	<0.001 **
**Utilization of antenatal care services at health center**				
Not utilized	14(3.1)	**1**		
Utilized	444(96.9)	0.5	0.3-0.9	0.02 *

**Notice**: (*) Indicates the statistical significance level at 0.05; (**) Estimates the statistical significance level at 0.01; Unmarked p-values were not fond to be the significant risk factors of LBW in Rwanda

### Multiple logistic regression analyses of the effects of the explanatory variables on birth weight of the newborns in Rwanda

Before conducting multivariate logistic regressions, VIF was checked and then the variables including weight, blood sample taken during the pregancy, urine sample taken during pregnancy, province of residence, provided anti-malaria drugs during pregnancy, wanted pregnancy when get pregnant, pressure taken during pregnancy, blood pressure taken and height inflacted the LBW since they scored more than 3 while the remaing variables remained with scores near to 1. Then, we removed the inflacted variables from the models. Therefore, VIF were reduced significantly and satisfactory. The model showed that babies from the mother who attended less than 4 ANC visits were almost three-times more likely to experience LBW [aOR=2.8; 95%CI (1.5-5.4), p=.002] compared to those whose mother attended 4 ANC visits. For marital status, bebing married and cohabiting pregnant women were significantly associated with an increase of the risk to have LBW babies [aOR=1.9; 95%CI (1.3-2.8), p=0.002], widow, divorced and separated from their husbands were significantly associated with an increase of having LBW babies [aOR=1.6 ; 95%CI (1.1-2.5), p=0.015] compared to single mothers during pregancy. Further, being provided maternal iron and folic acid supplements during pregnancy [aOR=0.5, 95%CI (0.3-0.9), p=0.015], having gestational age of ≥37 weeks [aOR=0.8, 95%CI (0.6-0.9), p<0.012], being informed about maternal complications of pregnancy [aOR=0.4, 95%CI (0.3-0.7), p=0.001] were associated with a decrease of LBW babies. Also, women with anemia [aOR=2.4, 95%CI (1.3-4.5), p<0.001] were positively associated with LBW. Through the number of the pregnant women who smoked during pregnancy, smoker birth weight is significantly associated with smoking, as pregnant women who did not smoke were less likely [aOR=0.3; 95%CI (0.1-0.6), p=0.002] to deliver LBW babies than their counterparts. Finally, the females babies were more likely to experience LBW [aOR=3.5, 95%CI (1.5-9.2), p=0.012] than the males babies ( Table 3).

**Table 3.** Multivariate logistic regression for the risk factors associated with low birth weight.

Characteristics	Low birth weight	Adjusted odds ratio		
	Percent		95% CI	*p-value*
**Type of residence**				
Urban	16.4	**1**		
Rural	83.6	1.1	0.7-1.6	0.008 *
**Sex of newborn**				
Males	44.3	1		
Females	55.7	3.5	1.5-9.2	<0.01 **
**Maternal education**				
Illiterate	15.1	**1**		
Primary/vocational	73.1	1.01	0.7-1.5	<0.001 **
Secondary/higher	11.8	0.99	0.6-1.8	<0.001 **
**Maternal age**				
15–19	2.6	**1**		
20–34	74.7	0.8	0.4-1.5	0.428
35–49	22.7	0.8	0.4-1.5	0.467
**Provided Tetanus injection during pregnancy**				
No	10.7	**1**		
Yes	89.3	0.3	0.1-0.7	0.021 *
**Body Mass index (BMI)**				
≤18.5 kg/m ^2^	80.2	**1**		
18.5-25.9kg/m ^2^	13.1	0.9	0.4-2.3	0.008 *
≥25.9kg/m ^2^	6.7	0.5	0.2-1.1	0.005 *
**Gestational age**				
<37 weeks	40.8	1		
≥37 weeks	59.1	0.8	0.6-0.9	0.012 *
**Smoking during pregnancy**				
Yes	3	**1**		
No	97	0.3	0.1-0.6	0.002 *
**Utilization of Antenatal care services**				
4 ANC and more	34.5	**1**		
No and less than ANC	65.5	2.8	1.5-5.4	0.002 *
**Parity for pregnant women**				
1-2	10.7	**1**		
3-4	73.8	2.7	1.1-6.7	0.052
≥5	15.5	2.3	0.9-5.5	0.09
**Family size**				
Less than 6	64.8	**1**		
6 members and more	35.2	1.02	0.8-1.4	0.918
**Household Wealth Index**				
Poor	51.5	**1**		
Middle/rich	48.5	0.9	0.6-1.4	0.728
**Anemia**				
Not anemic	43.9	1		
Anemic	56.1	2.4	1.3-4.51	<. 001 *
**Marital status**				
Single	15.5	**1**		
Married/cohabited	75.5	1.9	1.3-2.8	0.002 *
Widowed/separated/divorced	9.0	1.6	1.1-2.5	0.015 *
**During pregnancy, provided counseling about nutrients**				
Yes	55	**1**		
No	45	1.7	0.8-3.5	<0.003 *
**Provided tetanus injection during pregnancy**				
No	10.7	**1**		
Yes	89.3	0.3	0.8-2	0.337
**Received Iron and folic acid sumplements**				
No	20.8	**1**		
Yes	79.2	0.5	0.3-0.9	0.015 *
**Told about complications during pregnancy**				
No	18.9	1		
Yes	81.1	0.4	0.3-0.7	0.01 **
**Household head**				
Male	46.6	**1**	1	
Female	53.4	1.1	0.8-1.6	0.519

**Notice**: * Indicates statistical significance at p-value <0.05; ** Indicates significance at 0.01, Unmarked p-values were not fond to be the significant risk factors of LBW in Rwanda

## Discussion

Our study investigated the influence of ANC contacts on birthweight and their factors contributing to LBW babsies in Rwanda using RDHS. Interestingly, our results revealed that babies from the women who attended four and more ANC visits were less likely to have LBW than the babies from the pregnant women who attennded less than 4 ANC contacts; however, Rwanda reports a low prevalence of attending 8 recommended ANC contacts.These results were coincided with the previous studies that documented an improvement of ANC services contributing to birth weight of the newborns ^32^. Our results revealed a prevalence of 5.8% for LBW and 17.6% for macrosomia. The previous studies documented that the prevalence of LBW in Rwanda is low when compared with other counties in the same region such as Zambia, Uganda and Nigeria ^47– 49^. This magnitude is also less than the prevalence of some countries in Asia such as in China where the accessibility to ANC visits remains challenging ^50^. The present prevalence of LBW is less than the previous document which was 7.1% and low access of four recommended ANC visits was 35.4% ^51^. There is a significant reduction of the prevalence of LBW in Rwanda through the efforts made by the goverment whoen coumpared to the other developping countreis that has a prevalence of 15.9% ^6, 50, 52^.

Moreover, women who attended nutritional counseling were found to have a protective effect against LBW in this study, which is supported by previous studies ^53, 54^. This is because nutritional counseling may have a positive impact on the improvement of feeding behaviors of pregnant women and their nutritional status which may significantly contribute to a dicrease of the risk of delivering babies with LBW. Also, pregnant women who were fluently explained about pregnany complications were less likely to deliver LBW babies when compared to their counterparts. These findings are consistenct with the previous studies that indicated that ANC visits are significant contributor to birth weight, since it normalizes the birth weight of the child and their mother due to the prevention, intervention and health education that are effectively provided in each visit ^50, 55, 56^. Our results indicated that experiencing gestational age of more than 37 weeks reduced the risk for LBW babies. Further, non-smokers women and non-anemic women were at lower level to deliver LBW babies when compared to their counterparts. These results were relevant to prior studies conducted in central African counties that indicated that the attendance of ANC visits for pregnant women reduce the risk for delivering LBW babies. These findings are relevant to the previous studies that documented that smoking is a harmful behavior for the pregnant women that increase the risk for LBW babies ^25, 57– 59^ Further, gestationa age of more than 37 weeks was significantly associated with a reduction of the LBW babies. These results agreed with the earlier studies ^21, 53, 60^. These results were similar to the previous studies that documented that children born to educated pregnant women and pregnant women with high wealth index who attend are less likely to weigh less than 2.5kg than other pregnant women ^30, 61^. Prominently, in this research, the results are robust to adjustment for the socio-demographic characteristics of the recruited pregnant women. They revealed that marital status increased the odds of LBW. It was found that pregnant women who were married or living with the cohabitants as well as divorced and separated from their partner were more likely to deliver LBW newborns than the women who were single during pregnancy. These results challenged the previous studies that indicated being single was significantly associated with a greater risk of having LBW children than others ^48, 62, 63^.

Newborns whose mothers were had normal BMI (18.5–24.9 kg/m ^2^) and mothers who were overweight (or obese;) were less likely to have new-born with LBW when compared to those mothers who were underweight. This result is consistent with the earlier research carried out in Uganda ^64^. Newborns of women who smoke during pregnancy had low birth weight when compared with babies whose mothers did not smoke during pregnancies. Our findings are in convenance with the studies conducted in Tanzania ^65^. Mothers from rural areas had greater odds to deliver LBW babies than those residing in urban settings. Having secondary and higher education levels for pregnant women was significantly associated with a dicrease of LBW newborns than having information education. In agreement with previous studies ^63, 65^, women who were provided iron and folic acid sumplements during pregnancy were at lower risk to deliver LBW babies. This lower risk because iron-alone sumpplement could be protective factor against the LBW as compared to the diverse micronutrients sumpplementation and this sumplement has a significant contribution to a decline in the prevalence of intrauterine growith retardation with folic acid supplementation as it was previously documented by the previous researchers ^39, 65^.

Maternal anemia was identified as a risk factor, where we found that pregnant women with anemia had greater odds of delivering a newborn who weighed less than 2.5 kg than those who are not anemic. This is because moderate to severe anaemia for pregnant women could not only affect the mother but also impair oxygen delivery to the fœtus and thus interfere with normal intrauterine growth. These results are similar to the findings from the previous studies that reported that micronutrients defisciency during pregnacy has harmful impact on the development of fœtus and hence, birth weight. This result is in line with the prior studies conducted in Nigeria ^48, 60^. Females babies were more likely than males. These findings were supported by previous study conducted in Ethiopia ^66^. Although several socio-demograpghic factors, nutritional factors, behavioral factors and ANC contacts including maternal age, parity, HWI and family size were not significantly associated with the LBW in our study; however, the previous studies conducted in sub-Saharan African countries peviosuly reported them as the risk factors of LBW ^48, 66^.

## Strengths, limitations and future directions

The present research has numerous prominent strengths. The first strength is that the RDHS 2014/2015 used the validated and standardized tools for interviewing the eligible participants. Second, the research data about birth weight that we used were obviously verified through records and recall bias was prevented. Another important strength is that the findings from this study have a primordial implication for preventing LBW and inform the policy makers to promote nutritional education or counseling, provision of the sumplements namely iron-folic acid and prevention of anemia among the pregnent women. However, several limitations also warrant discussion. First, it was limited to a study design that did not actually explore for modifiable factors of ANC visits like HIV status of pregnant women, prenatal depression, receiving the supplementary vitamins (like vitamin A) during the pregnant, ultrasound, eclampsia, gestational diabetes, high blood glucose level during pregnancy, antiretroviral (ART) for HIV-positive women and reducing transmission of HIV from pregnant mother to child. Second, birth weight data was available for only babies whose weight was measured at birth. This limited us for estimating the real prevalence. Third, the selection bias resulted in the underestimation of the association between ANC and birth weight. This was because not all babies’ weight was measured. The other limitation was that the qualitative aspects data from the participants was not included for exploring some factors and to triangulate the findings of the quantitative methods used. Additionnally, as the attendance of 8ANC visits required by 2016 WHO was very low (less than 1%), it was impossible to categorise the ANC visits as an outcome variables respecting such recommendation. Indeed, the qualitative nature of the risk factors captured in the RDHS, like iron intake and duration, nutrition intake, maternal anemia and malnutrition status, which influence the birth weight. Additionally, as the methods used for measuring birth weight were not validated by the survey team, the misclassification could occur in this research. As we do not know the exact timing of the birth weight measurement, this causes misclassification. Further, our study design could not determine causal relationship; so, further research on longitidinal study design are needed.

## Conclusion

In conclusion, this study found several risk factors of LBW in Rwanda that remains a national public health concern. However, ANC services for pregnant women are a fundamental intervention for reducing LBW, the pregant women of Rwanda are encouraged to attend all 8 recommended ANC visits for increasing the quality of their life and the life of the babies. This integral part of primary health care, ANC services, is the population-based intervention that reduces the risk to deliver the newborn with LBW. Therefore, there is a need of a multifaceted approach for addressing the factors of LBW through reinforcing ANC coverage and quality of ANC services for pregnant women. Information on anthropometric measurement and increased awareness of the importance of regular ANC visits is also desirable. Attending four or more ANC visits is a step toward improving maternal health by providing maximum required packages and this strategy reduces the risk to LBW. The policy makers and researchers should prioritize maternal education and families with low socio-economic status for preventing LBW in Rwanda. We recommend further research be conducted on the risk factors of macrosomia in Rwanda. Additionally, further study to explore the barriers to access ANC visits effectively for pregnant women from the rural settings is recommended. In the meantime, pregnant women in Rwanda are required to attend ANC visits appropriately and regularly for benefiting from the required packages that increase the probability to attenuate the LBW among the newborns. We recommend to measure birth weight immediately after birth for reducing the recall bias and misclassification.

## Ethics and consent to participate

The present study was exempt from searching the ethical approval because the RDHS 2014/15 obtained the ethical approval from the Institutional Review Board (IRB) of the Rwanda National Ethics Committee (RNEC).

## Data Availability

Underlying data for ‘Regular antenatal care visits were associated with low risk of low birth weight among newborn in Rwanda: Evidence from the 2014/2015 Rwanda Demographic Health Survey (RDHS) Data was owned by the DHS program that can be obtained from https://dhsprogram.com/methodology/survey/survey-display-468.cfm. The electronic data is available from the DHS program under its terms of use. Before downloading the data, the main author of this study registered as DHS user for reasons laid out on the DHS program website and dataset access was only granted for legitimate purpose of this research.
