# Supplementary material for: Regular antenatal care visits were associated with low risk of low birth weight among newborns in Rwanda: Evidence from the 2014/2015 Rwanda Demographic Health Survey (RDHS) Data
Source: F1000Res. 2022 Nov 3;10:402. Originally published 2021 May 19. [Version 2] doi: 10.12688/f1000research.51969.2 (PMC9641107; doi:10.12688/f1000research.51969.2)
Supplement: Peer review history

# Peer review: Reviewer response for version 2

**DOI:** 10.5256/f1000research.139674.r154974

**Published:** 2022-11-07

**Authors:** M. Mazharul Islam

**Affiliations:** 1 Department of Statistics, College of Science, Sultan Qaboos University, Muscat, Oman

No further comment.

Is the work clearly and accurately presented and does it cite the current literature?

Yes

If applicable, is the statistical analysis and its interpretation appropriate?

Partly

Are all the source data underlying the results available to ensure full reproducibility?

Yes

Is the study design appropriate and is the work technically sound?

Partly

Are the conclusions drawn adequately supported by the results?

Partly

Are sufficient details of methods and analysis provided to allow replication by others?

Yes

Reviewer Expertise:

NA

I confirm that I have read this submission and believe that I have an appropriate level of expertise to confirm that it is of an acceptable scientific standard.

# Peer review: Reviewer response for version 1

**DOI:** 10.5256/f1000research.55185.r142748

**Published:** 2022-08-12

**Authors:** Sarmila Mazumder

**Affiliations:** 1 Society for Applied Studies, New Delhi, India

This is an important domain and the paper reiterates the importance of antenatal care. However, this a rather simplistic view of a complex issue. Few points to consider: 

1. The focus of the manuscript is on the number of ANC visits and not on the quality, content, examination, investigations, ultrasound, or respectful care, ability to identify/manage or refer high risk pregnancies, etc. The underlying assumption is that these are optimum at the the ANC sources. In that case, information on the same needs to be provided.
2. Additionally, just the number of ANC visits is not enough, gestational age when the pregnant women was first registered, the timeliness of the ANC visits going by the 4 ANC norm, that is, one in the first trimester, 1 in the second trimester and 2 in the third trimester, are equally important.
3. The ANC standard needs mention, there are women with 7 ANCs. Is the 2016 WHO ANC guideline being implemented at the study site? In that case is the rationale of 4 ANC as a cut-off based on minimum ANC visits? Need clarity.
4. Specific issues: How were the 8004 women selected? It is mentioned that that women who had documented birth weight were interviewed. The sampling method says 2 stage cluster sampling and random selection. In that case the selection of women seems purposive, this needs clarity.
5. This is a retrospective data collection, where women with youngest child less than 5 years were interviewed. It will be important to mention that all records on birth weight, number of ANC visits, measurement of BP, administration of antimalarial drug, height, weight and BMI, etc were available. Recall bias cannot be ruled out unless good documentation was available.
6. At which stage was BMI measured? Pre-pregnancy or early first trimester? This will be important to document.
7. Gestational weight gain is an essential component of ANC, this is not specified.
8. It is not clear why BP measurement and antimalarials are considered as confounders. 
9. Tables do not have N (total number, denominators). Table 1 needs explanation. The sample size was 8004 women but in table 1, the numbers do not add up to 8004. it varies with each variable. There is no information on the number of births. It would be informative to know about preterms, SGA, AGA as well.
10. The information on availability of birth weight is a little confusing. 92% reported birth weight, were these documented on discharge reports or verbally reported. Additionally, the low birth weight does not seem to be a major issue, with only 5.8%  low birth weight while 18% macrosomia. 
11. Some information would be useful to present. 99% women attended ANC clinic but 44% had 4 ANCs. A qualitative component would have been helpful to understand the barriers to low utilization.
12. The discussion section needs strengthening, it is repetition of the previous section. Some of the findings could have been analyzed, for eg., explore further how was home delivery associated with LBW. The places of ANC, pros and cons, etc. have  not been discussed. 
13. Typographical error, table 2, 7th row, last column. 0.0.3.
14. The authors need to match the numbers in the tables and text. 

Is the work clearly and accurately presented and does it cite the current literature?

Yes

If applicable, is the statistical analysis and its interpretation appropriate?

Partly

Are all the source data underlying the results available to ensure full reproducibility?

Yes

Is the study design appropriate and is the work technically sound?

Partly

Are the conclusions drawn adequately supported by the results?

Partly

Are sufficient details of methods and analysis provided to allow replication by others?

Partly

Reviewer Expertise:

Maternal and child health

I confirm that I have read this submission and believe that I have an appropriate level of expertise to confirm that it is of an acceptable scientific standard, however I have significant reservations, as outlined above.

# Response

**Published:** 2022-10-10

**Authors:** Emmanuel Biracyaza

**Affiliations:** University of Rwanda, Rwanda

This is an important domain, and the paper reiterates the importance of antenatal care. However, this a rather simplistic view of a complex issue. Few points to consider:

**Response: Dear reviewer, thank you very much for these kind words and crucial suggestions and recommendations for improving our study that was submitted in the f1000research.com. All the point you raised so that we can improve our study have been relevant and they helped us to continue looking our further investigation. Frankly, the ANC contacts remains at a very low level (less than 1%) which represents pregnant women who attended 8 ANC contacts. This is similar to the other countries of the same region (SSA) or LMICs.**

 The focus of the manuscript is on the number of ANC visits and not on the quality, content, examination, investigations, ultrasound, or respectful care, ability to identify/manage or refer high risk pregnancies, etc. The underlying assumption is that these are optimum at the ANC sources. In that case, information on the same needs to be provided.

**Responses: Thank you for the recommendations that are important. We totally agree with you about the focus of this work. But these variables about ultrasound, respectful care and risk pregnancies etc. were not found in the RDHS and we cannot change the dataset. But we recommend that the further studies can investigate and include such variables. In our data analysis, we included socio-demographic, nutritional variables, maternal and individual variables. But we did not find that the information about ultrasound or respectful care etc., however, we know how they are important in the ANC contacts.**

 Additionally, just the number of ANC visits is not enough, gestational age when the pregnant women was first registered, the timeliness of the ANC visits going by the 4 ANC norm, that is, one in the first trimester, 1 in the second trimester and 2 in the third trimester, are equally important.

**Responses: We thank you for this essential observation and suggestion. We have checked and corrected according to the suggestions provided. Variables such as gestational age were added. But about the ANC visits, we found that majority of the attended 4-7 ANCs while only 1% attended 8 recommended ANC contacts by the 2016 WHO but we have recommended the decision makers to encourage the pregnant women to attend the ANC visits as recommended by WHO. The main challenge was that the utilisation of 8 ANC visits is at low level and this could limit us in the data analysis.**

 The ANC standard needs mention, there are women with 7 ANCs. Is the 2016 WHO ANC guideline being implemented at the study site? In that case is the rationale of 4 ANC as a cut-off based on minimum ANC visits? Need clarity.

**Responses: The cut-off of used is the 4 ANC visits as recommended by the WHO due to the evidence provided above. We wrote an unintentional in our previous version and this created the mismatch in the first version. We notice that the attendance of the ANC as required remains a public health concern in Rwanda as one of SSA countries which remain with such concern that has to be solved through multidisciplinary team.**

 Specific issues: How were the 8004 women selected? It is mentioned that that women who had documented birth weight were interviewed. The sampling method says 2 stage cluster sampling and random selection. In that case the selection of women seems purposive, this needs clarity.

**Responses: Thank you as the study used secondary study (dataset from DHS), we did not conduct the data collection with direct contact with the women. But the RDHS indicates that the women were interviewed. The sample size was clearly indicated (7381 women who fit the inclusion criteria). We only included the women who provided information about the birth weight (they were only 7381) while 8004 was for the women who gave birth. Of course, if we take 8004-7381, we directly find the number of those who did not provide birth weight for their babies.**

 This is a retrospective data collection, where women with youngest child less than 5 years were interviewed. It will be important to mention that all records on birth weight, number of ANC visits, measurement of BP, administration of antimalarial drug, height, weight and BMI, etc were available. Recall bias cannot be ruled out unless good documentation was available.

**Response: Thank you for this recommendation that has helped us to improve our study. We included your recommendations and clarified the above-mentioned part.**

 At which stage was BMI measured? Pre-pregnancy or early first trimester? This will be important to document.

**Response: This was documented in the study. BMI as an important anthropometric indicator for pregnant women and can have a crucial effect on weight of child was measured at early first trimester (when the pregnant women attended the 1** ^st^ **ANC contact).**

 Gestational weight gain is an essential component of ANC, this is not specified.

**Responses: Thank you. The gestational age was added in the analysis; however, gestational diabetes was not included because of having several missing information.**

 It is not clear why BP measurement and antimalarials are considered as confounders.

**Responses: We have corrected according to your recommendations. By analysing the multicollinerality, we found that the variables such as variables related to malaria and BP inflacted with the birth weight. As results, we removed them from the analysis and we remained with the good variables that presented the variables, which seemed to be equal (near to 1). But the removed ones had more than 3 values of VIF.**

 Tables do not have N (total number, denominators). Table 1 needs explanation. The sample size was 8004 women but in table 1, the numbers do not add up to 8004. it varies with each variable. There is no information on the number of births. It would be informative to know about preterms, SGA, AGA as well.

**Responses: All the tables were appropriately corrected. The mismatching was because we shared the wrong table. This impact in the interpretations. About the above variables such as AGA, preterms, and SGA; they had several missing variables. So, that is why we did not include them in the analysis.**

 The information on availability of birth weight is a little confusing. 92% reported birth weight, were these documented on discharge reports or verbally reported. Additionally, the low birth weight does not seem to be a major issue, with only 5.8% low birth weight while 18% macrosomia.

**Responses: Thank you very much for this but for the developing country like Rwanda, the prevalence of about 6% for LBW may be a public health burden if the further strategies to promote health for the pregnant woman and the newborns.**

 Some information would be useful to present. 99% women attended ANC clinic but 44% had 4 ANCs. A qualitative component would have been helpful to understand the barriers to low utilization.

**Responses: Thank you for these important recommendations. The qualitative study design was recommended based on our results. This can be found in the strengths and further directions.**

 The discussion section needs strengthening, it is repetition of the previous section. Some of the findings could have been analyzed, for e.g., explore further how was home delivery associated with LBW. The places of ANC, pros and cons, etc. have not been discussed.

**Responses: The discussion was also modified and of course improved due to your recommendations.**

 Typographical error, table 2, 7th row, last column. 0.0.3.

**Responses: This typo error and grammatical errors were corrected.**

 The authors need to match the numbers in the tables and text.

**Responses: We kindly thank you for these recommendations. We have matched the results from the tables and texts.**

 Thank you very much.

# Peer review: Reviewer response for version 1

**DOI:** 10.5256/f1000research.55185.r144427

**Published:** 2022-08-04

**Authors:** Manoja Kumar Das

**Affiliations:** 1 The INCLEN Trust International, New Delhi, Delhi, India

The article presents the prevalence of LBW and the risk factors for the same in Rwanda using the RDHS 2014/15 data.

 The manuscript needs attention in some sections:

 1. Methods

 1.1 Macrosomia definitions should be mentioned 

 2. Results

 2.1 The following statements need review and clarification. 

 "For marital status, married and cohabiting pregnant women were 1.86-times at greater risk to have LBW children [aOR=1.9; 95%CI (1.3–2.8), p=0.002] compared with single pregnant women."

 "The results indicated that the widowed, separated and divorced pregnant women had 1.7-times greater risk of having LBW babies [aOR=1.7; 95%CI (1.1–2.5), p=0.015] than women who were single during pregnancy."

 The two statements are in conflict. Please check.

 2.2. The statement "Results found that being provided maternal iron and folic acid supplementation during pregnancy [aOR=0.5, 95%CI (0.3–0.9), p=0.015], not being given nutrients during pregnancy [aOR=2.5, 95%CI (2-8.4), p<0.001], being told about maternal complications during pregnancy [aOR=0.7...." 

 How was the nutrient given assessed?

 3. Discussion 

 3.1. Please check the statements and discuss 

 "They revealed that marital status increased the odds of LBW. It was found that pregnant women who were married or living with the cohabitants were 1.9-times likely to have LBW compared with pregnant women who were single. They also revealed that the pregnant women who divorced or separated had 1.7-greater risks to have new-borns who weigh less than 2.5 kg."

 This statement needs to be examined carefully, whether the factor responsible was crowding, low SES and undernutrition or hunger.

 4. Limitations

 4.1 The limitations should mention the qualitative nature of the risk factors captured in the RDHS, like iron intake and duration, nutrition intake, maternal anemia and malnutrition status, which influence the birth weight.

Is the work clearly and accurately presented and does it cite the current literature?

Yes

If applicable, is the statistical analysis and its interpretation appropriate?

Yes

Are all the source data underlying the results available to ensure full reproducibility?

Partly

Is the study design appropriate and is the work technically sound?

Yes

Are the conclusions drawn adequately supported by the results?

Partly

Are sufficient details of methods and analysis provided to allow replication by others?

Yes

Reviewer Expertise:

Child health, public health

I confirm that I have read this submission and believe that I have an appropriate level of expertise to confirm that it is of an acceptable scientific standard, however I have significant reservations, as outlined above.

# Response

**Published:** 2022-10-10

**Authors:** Emmanuel Biracyaza

**Affiliations:** University of Rwanda, Rwanda

Reviewer 2

**First of all, I thank you for the valuable points you raised for us. They have of course and prominently helped us to strengthen our study.** 

 The article presents the prevalence of LBW and the risk factors for the same in Rwanda using the RDHS 2014/15 data.

**Response: Thank you very much for this appreciation.**

 The manuscript needs attention in some sections:

 1. Methods

 1.1 Macrosomia definitions should be mentioned

**Responses: Thank you very much for the recommendations. The concept macrosomia was also defined in the first paragraph of the background of the study. It was defined as when a baby is born with more than 4 kilograms (or 4000 grams).**

 2. Results

 2.1 The following statements need review and clarification.

 "For marital status, married and cohabiting pregnant women were 1.86-times at greater risk to have LBW children [aOR=1.9; 95%CI (1.3–2.8), p=0.002] compared with single pregnant women."

**Response: This sentence was rephrased and improved.**

 "The results indicated that the widowed, separated and divorced pregnant women had 1.7-times greater risk of having LBW babies [aOR=1.7; 95%CI (1.1–2.5), p=0.015] than women who were single during pregnancy."

 The two statements are in conflict. Please check.

**Responses: This sentence was rephrased and improved**

 2.2. The statement "Results found that being provided maternal iron and folic acid supplementation during pregnancy [aOR=0.5, 95%CI (0.3–0.9), p=0.015], not being given nutrients during pregnancy [aOR=2.5, 95%CI (2-8.4), p<0.001], being told about maternal complications during pregnancy [aOR=0.7...."

 How was the nutrient given assessed?

**Responses: This statement was also improved after adjusting some analyes due to the required analysis from the other reviewers.**

 3. Discussion

 3.1. Please check the statements and discuss

 "They revealed that marital status increased the odds of LBW. It was found that pregnant women who were married or living with the cohabitants were 1.9-times likely to have LBW compared with pregnant women who were single. They also revealed that the pregnant women who divorced or separated had 1.7-greater risks to have new-borns who weigh less than 2.5 kg."

**Responses: In discussion section, we improved this part, and we changed the statement as recommended by the reviewer.**

 This statement needs to be examined carefully, whether the factor responsible was crowding, low SES and undernutrition or hunger.

**Responses: Thank you very much the factors such as SES and undernutrition (based on wasting, underweight, stunting and obesity) were also explored.**

 4. Limitations

 4.1 The limitations should mention the qualitative nature of the risk factors captured in the RDHS, like iron intake and duration, nutrition intake, maternal anemia and malnutrition status, which influence the birth weight.

**Responses: Thank you very much for these recommendations; and we included the recommendation that further studies could also be conducted using qualitative methods for exploring the perspectives from the pregnant women about the factors that influence their attendance of ANC and the benefits from attending ANC services on child weight at birth. So, we believe that the women can be experts in this qualitative exploration.**

**Thank you very much for your prominent recommendations that have helped us to improve our study.**

# Peer review: Reviewer response for version 1

**DOI:** 10.5256/f1000research.55185.r98487

**Published:** 2021-11-22

**Authors:** M. Mazharul Islam

**Affiliations:** 1 Department of Statistics, College of Science, Sultan Qaboos University, Muscat, Oman

The paper addressed a very important issue of public health. The findings of the paper are likely to contribute in literature of the impact of antenatal care on birth weight. However, I have some observations, which are given below.

1. In the Introduction section, page 3, the authors stated that WHO recommended four or more ANC visits during pregnancy, which is a dated recommendation. Perhaps, the authors are referencing 2001 WHO recommended schedule of 4 ANC visits (Villar *et al.*, 2001). However, in 2016 WHO revised its recommendation and prescribed 8 or more visit schedules (see WHO (2016). WHO recommendations on antenatal care for a positive pregnancy experience. https://apps.who.int/iris/bitstream/handle/10665/250796/9789241549912-eng.pdf).
  The authors defined the main explanatory variable (no or less than 4 ANC and ≥ 4 ANC) based on the old schedule of ANC. Considering the low average frequency of ANC visits in Rwanda, I am not claiming that the analysis is wrong, but not mentioning the latest development is a clear oversight. The authors should discuss the compliance of 8 or more ANC visits in Rwanda. 
2. Under sampling design, page 4, the authors wrote:   ……. “current birth weights were not available for the most children”, which contradicts the last line of the paragraph stating that “It was found that 92% of newborns had a birth weight reported while 8% did not.”
3. The authors should provide clear information about the total number of births considered in the study, of whom birth weights were available for how many or what proportion of births. Among the children with birth weights, what proportion of birth weights was obtained from mothers' recall and what proportion from written information. What was meant by written information? Is it birth certificates or health cards?
4. The most important information which is missing in this study is the total sample size of the study population. In one place, the authors mentioned about 8,004 mothers of reproductive age, but the distribution of the mothers provided in column 2 of Table 1 does not reflect that. What are these numbers in column 2 of Table 1 and why there is a wide variation in number across the variables?
5. In page 6, under the Results section of Multiple logistic regression analysis: There are a lot of mistakes. Most of the aORs and the 95%CIs given within parenthesis do not correspond with the results presented in Table 3.
6. The discussion section has been written very poorly, as it is mostly a repetition of the description of the findings without providing any explanations or possible implications of their findings. To validate their results, the authors just wrote “the results are consistent or similar or corroborate with findings of other study” without providing any numerical evidence how their findings are consistent with other studies. 
7. There is a methodological problem in the application of multiple logistic regression analysis to the real data considered in this study. One of the basic assumptions of the regression model is that the explanatory variables are independent. However, in this study, all the selected explanatory variables are not independent. For example, maternal height and weight are linearly dependent, and they are also related to body mass index (BMI) because BMI = weight/height ^2^. Violation of the assumption of independence creates a serious problem in statistical analysis, which is known as multicollinearity problem, resulting in biased results. While applying regression model, the researchers should be careful about multicollinerity problem. The authors did not provide any information about the goodness of fit of the model and adequacy of the fitted model. The author should redo the multiple regression analysis by removing the suspected inter-correlated variables.     

Is the work clearly and accurately presented and does it cite the current literature?

Yes

If applicable, is the statistical analysis and its interpretation appropriate?

Partly

Are all the source data underlying the results available to ensure full reproducibility?

Yes

Is the study design appropriate and is the work technically sound?

Partly

Are the conclusions drawn adequately supported by the results?

Partly

Are sufficient details of methods and analysis provided to allow replication by others?

Yes

Reviewer Expertise:

NA

I confirm that I have read this submission and believe that I have an appropriate level of expertise to confirm that it is of an acceptable scientific standard, however I have significant reservations, as outlined above.

# Response

**Published:** 2022-10-10

**Authors:** Emmanuel Biracyaza

**Affiliations:** University of Rwanda, Rwanda

The paper addressed a very important issue of public health. The findings of the paper are likely to contribute in literature of the impact of antenatal care on birth weight. However, I have some observations, which are given below.

 In the Introduction section, page 3, the authors stated that WHO recommended four or more ANC visits during pregnancy, which is a dated recommendation. Perhaps, the authors are referencing 2001 WHO recommended schedule of 4 ANC visits (Villar et al., 2001). However, in 2016 WHO revised its recommendation and prescribed 8 or more visit schedules (see WHO (2016). WHO recommendations on antenatal care for a positive pregnancy experience. https://apps.who.int/iris/bitstream/handle/10665/250796/9789241549912-eng.pdf).

**Responses: Thank you very much; we found that there was an typing error since in our study, we used expected to use the recent recommendations of the WHO that recommend 8 ANC visits for the pregnant women. But when we realised that it could not be possible to assess it because the prevalence of using 8 recommended ANC contacts was low in Rwanda (less than 1%). So, in the study limitations and conclusions we tried to mention it and then recommend the policy makers to encourage pregnant women to respect the respected ANC contacts.**

 The authors defined the main explanatory variable (no or less than 4 ANC and ≥ 4 ANC) based on the old schedule of ANC. Considering the low average frequency of ANC visits in Rwanda, I am not claiming that the analysis is wrong, but not mentioning the latest development is a clear oversight. The authors should discuss the compliance of 8 or more ANC visits in Rwanda.

**Responses: Thank you very much. As indicated in the work, we considered the 8 ANC visits as recommended by 2016 WHO. So, the problem was in the first table where we shared the wrong table, but we effectively corrected it and then provided the appropriate table.**

**Notice. For the case of 2016 WHO recommendations, you can also refer to the following publications to indicate you how the ANC remains very low:**

 1. Sserwanja, Q., Nuwabaine, L., Gatasi, G. *et al.* Factors associated with utilization of quality antenatal care: a secondary data analysis of Rwandan Demographic Health Survey 2020. *BMC Health Serv Res* **22**, 812 (2022). https://doi.org/10.1186/s12913-022-08169-x

 2. Rurangirwa, A.A., Mogren, I., Ntaganira, J. *et al.* Quality of antenatal care services in Rwanda: assessing practices of health care providers. *BMC Health Serv Res* **18**, 865 (2018). https://doi.org/10.1186/s12913-018-3694-5

 Under sampling design, page 4, the authors wrote:   ……. “current birth weights were not available for the most children”, which contradicts the last line of the paragraph stating that “It was found that 92% of newborns had a birth weight reported while 8% did not.”

**Responses: We edited the sentence after noticing that what you have observed. We decided to remove it because we found it was not necessary. Thus, the last line of the paragraph was really enough.**

 The authors should provide clear information about the total number of births considered in the study, of whom birth weights were available for how many or what proportion of births. Among the children with birth weights, what proportion of birth weights was obtained from mothers' recall and what proportion from written information. What was meant by written information? Is it birth certificates or health cards?

**Responses: We thank you very much for this suggestion. Normally, after we recheck our dataset and remove missing variables, we also tried to indicate the corrected number of the births and pregnant women. Please, note that we only used the data of the women who provided the number weight of their newborns at birth. the women who were of out of this major inclusion criteria were removed from the analyses (therefore, our study was conducted among 7381 women).**

 The most important information, which is missing in this study, is the total sample size of the study population. In one place, the authors mentioned about 8,004 mothers of reproductive age, but the distribution of the mothers provided in column 2 of Table 1 does not reflect that. What are these numbers in column 2 of Table 1 and why there is a wide variation in number across the variables?

 Responses: Thank you very much for this observation about mismatching of the totals for the table 1. We included the corrected table. In addition to that, the sample size was 7381 women who provided the weight of their babies. They were recruited from 8004 women who delivered and 30058 individuals for the participants of this dataset in which we used.

 In page 6, under the Results section of Multiple logistic regression analysis: There are a lot of mistakes. Most of the aORs and the 95%CIs given within parenthesis do not correspond with the results presented in Table 3.

**Responses: We thank you or this observation. We have corrected the interpretations since the information provided in the table was correct. Improved interpretations of the results were also made.**

 The discussion section has been written very poorly, as it is mostly a repetition of the description of the findings without providing any explanations or possible implications of their findings. To validate their results, the authors just wrote “the results are consistent or similar or corroborate with findings of other study” without providing any numerical evidence how their findings are consistent with other studies.

**Responses: Regarding the discussion, we have importantly made the revisions and changes based on the major results from the section results.**

 There is a methodological problem in the application of multiple logistic regression analysis to the real data considered in this study. One of the basic assumptions of the regression model is that the explanatory variables are independent. However, in this study, all the selected explanatory variables are not independent. For example, maternal height and weight are linearly dependent, and they are also related to body mass index (BMI) because BMI = weight/height2. Violation of the assumption of independence creates a serious problem in statistical analysis, which is known as multicollinearity problem, resulting in biased results. While applying regression model, the researchers should be careful about multicollinerity problem. The authors did not provide any information about the goodness of fit of the model and adequacy of the fitted model. The author should redo the multiple regression analysis by removing the suspected inter-correlated variables.     

**Responses: Thank you for these recommendations. To manage the issues of methods, we did again the analysis part and we found the new results with some differences. So, we really have computed the goodness of fit for the multivariate logistical model. Multicolinearity was assessed using VIF and as a result several variables that inflacted were directly removed from models for avoiding overestimation or underestimations. We really found that the removed variables negatively impacted some variables because some of the remaining variables became significantly associated while others were not. But we observed crucial improvements.**
